# Genetic Variations in the M6A Modification Pathway as Potential Predictors of Imatinib Secondary Resistance in Gastrointestinal Stromal Tumors

**DOI:** 10.3390/jcm15062183

**Published:** 2026-03-13

**Authors:** Baijun Zhou, Zhenchong Chen, Gengyi Zhang, Shaoxing Guan, Wei Zhuang, Min Huang, Haibo Qiu, Xueding Wang

**Affiliations:** 1 Institute of Clinical Pharmacology, School of Pharmaceutical Sciences, Sun Yat-sen University, Guangzhou 510080, China; zhoubj8@mail3.sysu.edu.cn (B.Z.); zhanggy26@mail2.sysu.edu.cn (G.Z.); guanshx6@mail.sysu.edu.cn (S.G.); huangmin@mail.sysu.edu.cn (M.H.); 2 Department of Gastric Surgery, Sun Yat-sen University Cancer Center, Guangzhou 510060, China; chenzc@sysucc.org.cn; 3 Department of Pharmacy, Women and Children’s Hospital, School of Medicine, Xiamen University, Xiamen 361005, China; zhuangwei1333@xmu.edu.cn

**Keywords:** genetic variation, single nucleotide polymorphism, imatinib mesylate, gastrointestinal stromal tumor, m6A modification, progression-free survival

## Abstract

**Background/Objectives:** Imatinib mesylate (IM) is the first-line therapy for gastrointestinal stromal tumor (GIST). Emerging evidence suggests that genes involved in m6A modification, autophagy, and DNA damage repair pathways might contribute to IM secondary resistance and result in substantial inter-patient variability in treatment outcomes. However, influence of the genetic variations in these genes on IM-treated GIST remains unclear. **Methods:** A total of 172 GIST patients treated with IM in Sun Yat-sen University Cancer Center from 2014 to 2018 were enrolled. A 6-month landmark analysis was applied to specifically investigate secondary resistance, restricting the cohort to patients alive and progression-free at 6 months. Tag single-nucleotide polymorphisms (SNPs) in 54 evidence-based candidate genes involved in m6A modification, autophagy, and DNA damage repair pathways were selected and genotyped. Associations between SNPs and progression-free survival (PFS) were assessed using univariate Cox regression and Kaplan–Meier analyses with time zero reset to the 6-month landmark. Identified SNPs were further analyzed in multivariable Cox models adjusted for demographic and clinical factors. **Results:** During a median follow-up of 53.62 months (range, 7.60–129.77) as of October 2023, 39 progression events occurred. Univariate analyses identified 12 SNPs located in 9 genes associated with PFS. After adjustment for demographic and clinical covariates, 10 SNPs remained associated with PFS. Of these, seven variants were located in m6A pathway genes (*ALKBH5*, *METTL3*, *YTHDC2*, and *ZC3H13*), four of which were associated with shorter PFS (e.g., *YTHDC2* rs1833678 T > C, HR = 2.87, 95% CI: 1.18–7.03, *p* = 0.021) and three with longer PFS (e.g., *METTL3* rs1263793 A > G, HR = 0.40, 95% CI: 0.20–0.83, *p* = 0.014) of progression. A cumulative genetic risk score based on the identified m6A SNPs was associated with PFS (*p* < 0.001). Additionally, one SNP in autophagy and two in DNA damage repair pathways also remained associated with PFS after adjustment. **Conclusions:** Genetic polymorphisms in the m6A modification genes, along with variants in autophagy and DNA damage repair pathways, were associated with PFS in IM-treated patients who had achieved initial disease control. The cumulative risk score based on m6A pathway variants showed a strong association with PFS. These findings provide preliminary, hypothesis-generating evidence that genetic variations may contribute to inter-patient variability in outcomes and warrant further investigation as potential biomarkers in IM-treated GIST.

## 1. Introduction

Gastrointestinal stromal tumor (GIST) is a rare disease and part of the soft tissue sarcomas, derived from the mesenchymal tissue of the gastrointestinal tract, with an annual incidence of approximately 1.2 per 100,000 [1,2]. It is primarily driven by activating mutations in receptor tyrosine kinase genes such as *KIT* and *PDGFRA* [3,4]. Imatinib mesylate (IM), the first small-molecule targeted therapy developed through computer-assisted drug design, received FDA approval for GIST in 2002 with expanded indications [5]. IM therapy has dramatically improved survival outcomes, extending the median survival of GIST patients from 18 months to more than 70 months [1], and remains the first-line treatment. However, there is substantial inter-patient variability in treatment outcomes for GIST. Long-term IM therapy is frequently accompanied by the development of secondary resistance, ultimately leading to diminished therapeutic efficacy or disease progression.

Secondary resistance was observed in approximately 40% to 50% of patients within two years of treatment [6]. The molecular mechanisms underlying secondary IM resistance are not yet fully elucidated. Existing research mainly focused on somatic mutations. Existing evidence shows that secondary somatic mutations, particularly those involving *KIT* or *PDGFRA*, markedly reduce IM sensitivity. In a phase II clinical study enrolling 147 patients with advanced GIST, 67% of IM-resistant individuals acquired new secondary *KIT* mutations [7]. However, in clinical practice, GISTs with the same somatic mutation may also exhibit different responses, indicating that IM resistance may involve other mechanisms as well [8].

Germline genetic polymorphisms, as stable and heritable sources of inter-individual variation, represent important intrinsic factors likely to influence drug response. Accumulating evidence also suggests that the therapeutic efficacy of IM may be modulated by germline genetic polymorphisms. Angelini et al. studied 54 patients with unresectable/metastatic GISTs and found that *OCTN1* rs1050152 C > T, *OCTN2* rs2631367 C > G, and *OCTN2* rs2631372 C > G were associated with prolonged time to progression [8]. In another study involving 227 European GIST patients, Verboom et al. reported that *KDR* rs1870377 AA, *VEGFA* rs1570360 AA, and *SLCO1B3* rs4149117 GT/TT genotypes were associated with shorter progression-free survival (PFS) [9].

Meanwhile, recent studies suggest that various biological processes, such as epigenetic regulation, autophagy, and DNA damage repair may contribute to the development of IM resistance [10,11,12]. For example, Xu et al. showed that the m6A methyltransferase METTL3 enhances the translation of MRP1, driving IM resistance [13]. IM has been reported to stabilize ATG5 protein to enhance autophagy in GIST cells, thereby promoting drug resistance [10]. Regarding DNA damage repair, a case report described a rare BRCA2 mutation in GIST was identified in a Chinese patient who failed multiple lines of therapy including IM, suggesting a potential association between BRCA2 and IM resistance [14]. Collectively, these findings suggest that IM resistance is under multi-level and multi-pathway regulation, with genetic variations in key genes potentially serving as critical regulatory elements.

Based on these considerations, we conducted a genetic association study with extended follow-up, aiming to investigate whether germline polymorphisms in pathways previously implicated in IM resistance contribute to heterogeneous outcomes in IM-treated GIST. To specifically address secondary resistance, we restricted the primary analysis to patients who achieved initial disease control and assessed the association of selected tag single-nucleotide polymorphisms (SNPs) with PFS. We further evaluated the cumulative effect of risk variants within the m6A pathway, which emerged as the most consistently associated pathway in our analysis.

## 2. Materials and Methods

### 2.1. Patients and Study Design

This study is a genetic association analysis conducted in a cohort of patients who received the same treatment. All patients received continuous first-line imatinib monotherapy at the standard dose of 400 mg/day, as recommended by National Comprehensive Cancer Network (NCCN) guidelines [15]. The exposure variable of interest is the germline genotype, assessed within this consistent therapeutic background.

According to the NCCN guidelines, secondary resistance to imatinib in GIST patients typically occurs after more than 6 months of treatment, following an initial response or disease stability [15]. To specifically analyze secondary resistance and to mitigate immortal time bias, we employed a pre-specified 6-month landmark analysis. Our study cohort was therefore restricted to patients who were confirmed to be alive and free from radiologically defined disease progression at 6 months after treatment initiation. Disease progression was defined as the emergence of new lesions or an increase in tumor volume, as assessed by the treating clinicians based on Response Evaluation Criteria in Solid Tumors (RECIST) criteria [16].

This study was approved by the Ethics Committee of Sun Yat-sen University Cancer Center (Guangzhou, China). From 2014 to 2018, 172 GIST patients who were treated in Sun Yat-sen University Cancer Center, and met the inclusion criteria, with no exclusion criteria present, were enrolled. The inclusion criteria were as follows: (1) age between 18 and 80 years; (2) diagnosed with GIST confirmed by molecular diagnostics or pathology; (3) consistent use of first-line imatinib monotherapy (400 mg/day) for at least 6 months without progression; (4) voluntary participation with signed informed consent. The exclusion criteria included: (1) poor treatment compliance; (2) presence of other malignancies or uncontrolled systemic diseases (e.g., diabetes, advanced hepatic or renal failure); (3) combination of inhibitors or inducers of CYP3A4, CYP3A5, or P-glycoprotein (e.g., rifampin, digoxin, phenobarbital); (4) pregnancy or lactation.

Clinical information, including age, sex, and body surface area (BSA), as well as prognostic indicators recommended by the NCCN Guidelines [15], such as tumor size, mutation type and site, and mitotic index, were collected. BSA was calculated from patient height and weight data using a formula validated for the Chinese population: BSA (m^2^) = 0.0061 × height (cm) + 0.0124 × weight (kg) − 0.0099 [17]. PFS was used as the primary efficacy endpoint. All clinical assessments, follow-up data collection, and curative effects were conducted by doctors.

### 2.2. Candidate Gene Selection and Genotyping

Fifty-four candidate genes involved in the pathways of m6A modification, autophagy, and DNA damage repair were included. The gene list, with pathway classification and references, is provided in Supplementary Table S1. Peripheral whole-blood samples were collected from all enrolled patients. Genomic DNA was extracted using the TIANamp Blood DNA Kit DP348 (TIANGEN Biotech, Beijing, China). Tag SNPs within these genes were selected using Haploview (version 4.2, Broad Institute of MIT and Harvard, Cambridge, MA, USA) according to linkage disequilibrium patterns. Primers for multiplex PCR genotyping, including forward, reverse, and extension primers, were designed with the Assay Design Suite online tool (Agena Bioscience, Inc., San Diego, CA, USA). Genotyping was performed using the Agena MassARRAY system (Agena Bioscience, Inc., San Diego, CA, USA) based on the matrix-assisted laser desorption/ionization time-of-flight (MALDI-TOF) platform. DNA samples with a concentration of ≥30 ng/μL were used for the multiplex end-point PCR. The PCR products were spotted onto a chip to co-crystallize with the matrix and analyzed in a MALDI-TOF mass spectrometer to determine the nucleotide base [18,19].

### 2.3. Statistical Analysis

All analyses pertaining to secondary resistance were performed using a pre-specified 6-month landmark approach. Time zero was redefined as the 6-month time point after imatinib initiation, and PFS was calculated from this new time zero until disease progression, death, or last follow-up.

Hardy–Weinberg equilibrium (HWE) was assessed for the genotype frequency distribution of all successfully genotyped SNPs using the SNPStats online platform (Institut Català d’Oncologia, Barcelona, Spain; https://snpstats.net/, accessed on 15 March 2024). For the analysis of genetic variants, a dichotomized genotype grouping strategy was applied throughout the genetic association analyses.

In univariate analysis, the association between each variable and PFS was assessed using Cox proportional hazards regression to estimate hazard ratios (HRs) and 95% confidence intervals (CIs). For SNPs, Kaplan–Meier curves were generated and compared using the log-rank test. SNPs showing nominal significance in univariate analysis were considered for further adjustment. In multivariate analysis, a separate multivariable Cox proportional hazards model was constructed individually for each identified SNP in univariate analysis. The following demographic and clinical parameters were included as covariates in the model: age, sex, BSA, tumor location, maximum tumor diameter, and mitotic index. A cumulative genetic risk score was constructed based on risk variants within the m6A pathway and its association with PFS was evaluated using Cox regression.

Given the hypothesis-generating nature of this study, *p* values are reported without adjustment for multiple comparisons. All results should be regarded as preliminary findings that necessitate validation in future research.

Statistical analyses were conducted using Zstats (Zhejiang Chinese Medical University, Hangzhou, China; https://www.zstats.net/, accessed on 18 December 2025), and the forest plots were generated using the ‘forestplot’ package in R software (version: 2025.09.2+418, R Foundation for Statistical Computing, Vienna, Austria). The *p* value < 0.05 was considered statistically significant.

## 3. Results

### 3.1. Association Between Clinical Characteristics and PFS

By October 2023, a total of 172 patients receiving IM treatment were retrospectively enrolled in this study, with a median follow-up duration of 53.62 months, ranging from 7.60 to 129.77 months. By the end of follow-up, disease progression occurred in 39 patients, and 11 patients had died. At the last follow-up, the median PFS was not yet reached, as more than half of the patients (77.3%) remained progression-free. The 36-month PFS rate from treatment initiation was 85.6% (95% CI, 80.2–91.4%).

Demographic and baseline clinical characteristics, along with their univariate associations with PFS, are summarized in Table 1. The median age was 55 years, and the cohort comprised 79 (45.9%) females and 93 (54.1%) males. The median BSA was 1.71 m^2^. Regarding primary tumor site, 64.0% originated in the stomach, 25.6% in the small intestine, and 10.5% in other locations. Tumor somatic mutation testing (via biopsy or postoperative specimens) showed that 93 patients carried *KIT* exon 11 mutations, 7 had *KIT* exon 9 mutations, 1 had a *KIT* exon 17 mutation, and 20 patients were wild-type for both *KIT* and *PDGFRA*. The remaining 51 patients did not undergo somatic mutation testing due to a lack of surgical or biopsy samples.

Mitotic index > 5/50 high-power fields (HPF) was associated with shorter PFS (HR = 2.53, 95% CI: 1.19–5.38, *p* = 0.016). In contrast, age, sex, BSA, tumor site, tumor size, and tumor mutation status were not significantly associated with PFS.

### 3.2. Univariate Analysis of SNPs Associated with PFS

A total of 141 tag SNPs were selected from 54 candidate genes. Hardy–Weinberg equilibrium (HWE) testing was performed for tag SNPs. A total of 24 SNPs deviated from HWE and were excluded, whereas 117 SNPs were in HWE and included in subsequent association analyses. The list of selected SNPs and their HWE results is presented in Supplementary Table S2.

Log-rank tests and univariate Cox regression were conducted to evaluate the associations between each candidate SNP and PFS. In the m6A modification pathway, several SNPs showed associations with PFS (Table 2). For *ALKBH5* rs71372273 T > A and *YTHDC2* rs114925657 T > C, carriers of the rare homozygous genotypes (AA for rs71372273 and CC for rs114925657) exhibited substantially shorter PFS compared with reference genotype carriers (HR = 99.49, 95% CI: 13.72–721.52, *p* < 0.001; and HR = 56.50, 95% CI: 5.88–543.15, *p* < 0.001, respectively). In addition, *YTHDF2* rs7520756 C > T, *YTHDC2* rs1833678 T > C, *WTAP* rs2758313 C > G, and *ZC3H13* rs7318648 T > C were also associated with shorter PFS, with HRs ranging from 2.00 to 2.59 (all *p* < 0.05). Conversely, *METTL3* rs1139130 A > G and rs1263793 A > G and *ZC3H13* rs17703559 A > G were associated with longer PFS (HR < 1, *p* < 0.05). Furthermore, *ATG5* rs117827198 C > T in the autophagy pathway, along with *MBD4* rs140696 G > A and *ERCC6* rs4253231 A > G in the DNA damage repair pathway, were associated with shorter PFS.

**Figure 1:**
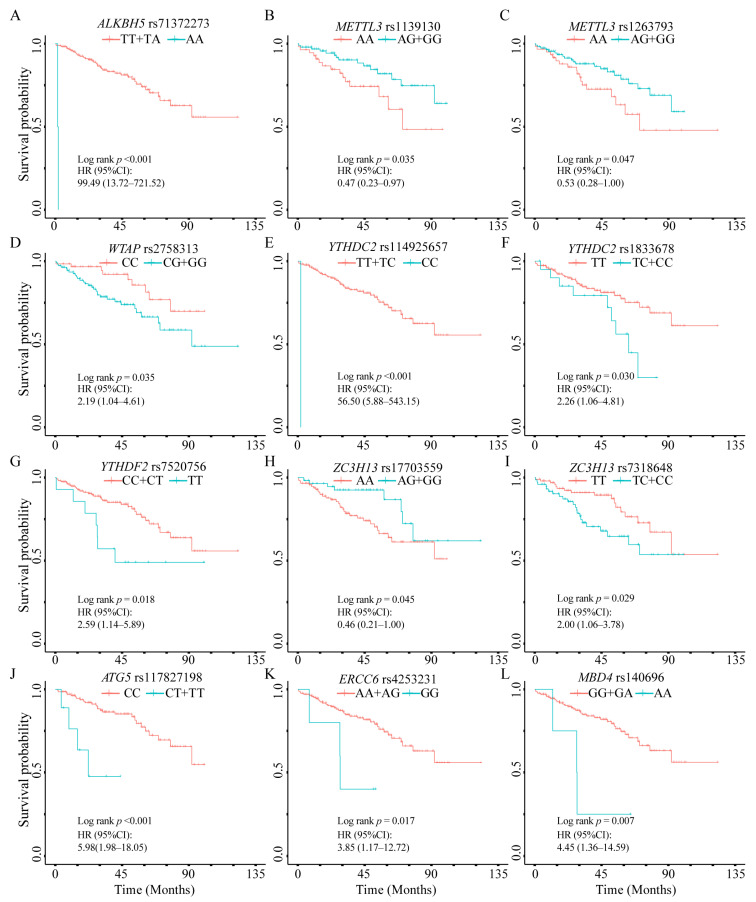
Kaplan–Meier curves for PFS according to genotype of selected SNPs in the 6-month landmark cohort. (**A**). *ALKBH5* rs71372273; (**B**). *METTL3* rs1139130; (**C**). *METTL3* rs1263793; (**D**). *WTAP* rs2758313; (**E**). *YTHDC2* rs114925657; (**F**). *YTHDC2* rs1833678; (**G**). *YTHDF2* rs7520756; (**H**). *ZC3H13* rs17703559; (**I**). *ZC3H13* rs7318648; (**J**). *ATG5* rs117827198; (**K**). *ERCC6* rs4253231; (**L**). *MBD4* rs140696.

Kaplan–Meier survival curves for SNPs associated with PFS are presented in Figure 1. SNPs showing nominal significance (*p* < 0.05) in univariate analysis were further evaluated in multivariable Cox regression models to assess their association with PFS after adjustment for clinical covariates.

Notably, several significant associations involved rare genotype subgroups with small sample sizes (<5 individuals). These analyses are exploratory and statistically underpowered. The findings should therefore be interpreted with caution and are presented primarily to generate hypotheses for future investigation.

### 3.3. Multivariate Adjustment Analysis of SNPs Associated with PFS

To assess whether each SNP was associated with PFS after adjustment for clinical covariates, multivariable Cox proportional hazards regression analyses were performed with adjustment for age, sex, BSA, tumor location, maximum tumor diameter, and mitotic index. The results are summarized in Table 3.

**Figure 2:**
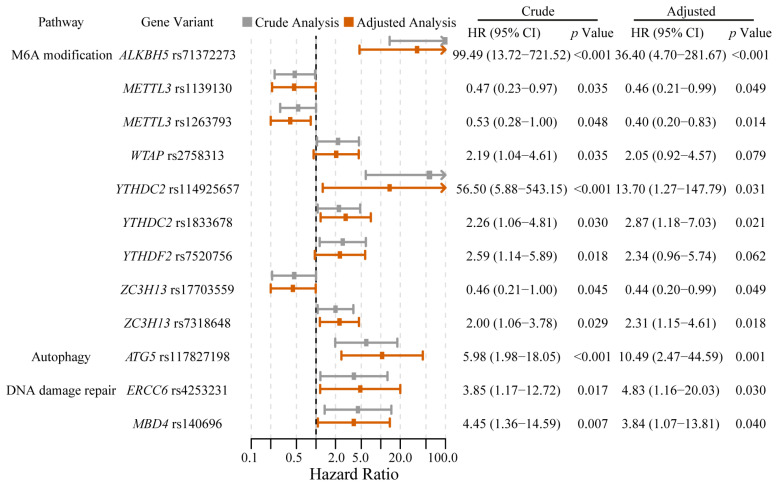
Forest plot of univariate and multivariable-adjusted associations of SNPs with PFS in the 6-month landmark cohort.

After adjustment for demographic and clinical covariates, 10 SNPs remained associated with PFS. *ALKBH5* rs71372273 T > A, *YTHDC2* rs114925657 T > C, and *ATG5* rs117827198 C > T showed strong associations with substantially shorter PFS, with adjusted HR of 36.40 (95% CI: 4.70–281.67, *p* < 0.001), 13.70 (95% CI: 1.27–147.79, *p* = 0.031), and 10.49 (95% CI: 2.47–44.59, *p* = 0.001), respectively. In addition, associations with shorter PFS were observed in *YTHDC2* rs1833678 T > C, *ZC3H13* rs7318648 T > C, *ERCC6* rs4253231 A > G, and *MBD4* rs140696 G > A with adjusted HRs ranging from 2.31 to 4.83 (all *p* < 0.05). Conversely, *METTL3* rs1263793 A > G and rs1139130 A > G and *ZC3H13* rs17703559 A > G were associated with longer PFS (adjusted HR < 1, *p* < 0.05). Although *YTHDF2* rs7520756 C > T and *WTAP* rs2758313 C > G showed elevated hazard ratios, their associations with PFS were not statistically significant after multivariable adjustment (*p* > 0.05). The univariate and multivariable-adjusted associations between SNPs and PFS are summarized in Figure 2.

### 3.4. Cumulative Effect of SNPs Within the m6A Pathway

Our prior analysis identified multiple SNPs within key regulators of the m6A pathway that were associated with PFS. To investigate the cumulative effect of these pathway-specific genetic variants, we constructed a genetic risk score (GRS). The GRS was calculated by assigning 1 point for each patient carrying the risk allele associated with shorter PFS for each SNP, and 0 points otherwise. Among the 149 patients with complete genotype data for 9 included SNPs, the observed GRS range was from 0 to 6 (theoretical range: 0–9). Based on this score, patients were stratified into three groups: low-risk (score 0–2, n = 65), intermediate-risk (score 3–4, n = 68), and high-risk (score 5–6, n = 16).

**Figure 3:**
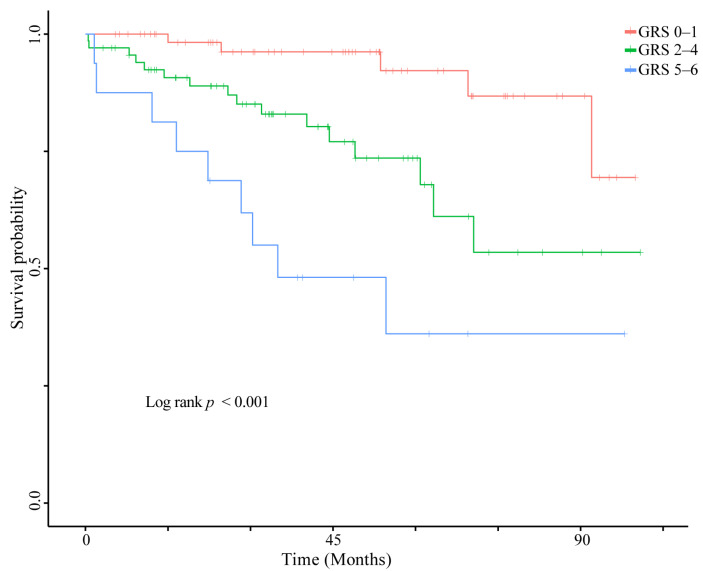
Kaplan–Meier curves of PFS according to the GRS based on m6A pathway SNPs in the 6-month landmark cohort.

The Kaplan–Meier survival curve showed PFS differed among the three risk groups (Log-rank *p* < 0.001; Figure 3). As shown in Table 4, in univariate analysis, both the intermediate-risk and high-risk groups had shorter PFS compared to the low-risk group (both *p* < 0.05). After adjustment for demographic and clinical covariates, this association persisted for both intermediate-risk group (HR = 3.40, 95% CI: 1.15–10.07, *p* = 0.027) and high-risk group (HR = 9.42, 95% CI: 2.57–34.44, *p* < 0.001). Furthermore, when the GRS was analyzed as a continuous variable, it remained associated with shorter PFS (HR per grade increase = 3.05, 95% CI: 1.62–5.75, *p* for trend < 0.001), suggesting a graded relationship between genetic risk burden and clinical outcome. In summary, these findings indicate a cumulative association of risk-associated SNPs within the m6A RNA modification pathway with PFS in this cohort.

## 4. Discussion

Substantial inter-patient variability in treatment outcomes and the frequent development of secondary resistance limit the long-term efficacy of IM in GIST patients. The current guideline-recommended strategy for secondary resistance includes escalating the IM dose or switching to second-line agents such as sunitinib. However, these interventions have a delayed onset of action, which may lead to a lag in treatment plans and pose a risk of delaying disease control. Therefore, there is a clear need for new biomarkers that can predict prognosis earlier or identify patients at high risk of drug resistance or disease progression. In this hypothesis-generating study, we investigated whether germline polymorphisms in pathways implicated in imatinib resistance are associated with secondary resistance in GIST patients who achieved initial disease control.

To focus on secondary resistance, we employed a pre-specified 6-month landmark analysis. This ensured all analyzed patients had surpassed the primary resistance phase, thereby refining our focus to the genetics of secondary resistance. Thus, the associations observed suggest a potential, phase-specific role for these variants in the development of resistance after initial clinical benefit. However, the landmark approach has an inherent limitation, which does not account for individual variation in the timing of initial benefit. For patients who responded earlier, the true biological interval from response to resistance may be underestimated. While this method mixes different durations of initial benefit, it provides a relatively robust and unbiased common starting point for comparison in a retrospective setting, as it systematically excludes early progressors and aligns with the clinical definition of secondary resistance.

In this study, we identified multiple SNPs in genes involved in m6A RNA modification, autophagy and DNA damage repair pathways that were associated with PFS in GIST patients who achieved initial disease control on imatinib. A total of 12 polymorphisms in *ALKBH5, METTL3, WTAP, YTHDC2, YTHDF2, ZC3H13, ATG5, ERCC6,* and *MBD4* showed associations with PFS in univariate analysis, and 10 of them remained associated after adjustment for clinical confounders (age, sex, BSA, tumor location, maximum diameter, and mitotic index). Additionally, for four SNPs, the statistical power was limited by very small sample sizes (n < 5) in one subgroup, resulting in wide confidence intervals and imprecise hazard ratio estimates. These findings should therefore be interpreted with caution; however, the magnitude of the observed effect sizes suggests potential biological signals that warrant further investigation.

Genetic variants in key regulators of m6A modification were associated with PFS. After multivariable adjustment, variants associated with shorter PFS exhibited HRs ranging from 2.31 to 36.40, while those associated with longer PFS had HRs between 0.40 and 0.46 (Table 3). *ALKBH5* rs71372273 and *YTHDC2* rs114925657 showed HRs exceeding 10, with extremely wide confidence intervals (e.g., 4.70–281.67), reflecting small subgroup sizes and imprecise estimates. These findings require particularly cautious interpretation. Variants associated with shorter PFS showed HRs ranging from 2.05 to 2.87, suggesting effect sizes of potential clinical relevance. *YTHDC2* rs1833678 and *ZC3H13* rs7318648 remained associated after adjustment with 2.87-fold and 2.3-fold higher hazard of progression in variant carriers, respectively, compared to reference groups. *METTL3* rs1263793, *METTL3* rs1139130, and *ZC3H13* rs17703559 were associated with longer PFS, with HRs ranging from 0.40 to 0.46, suggesting a potential protective effect. Although the confidence intervals were relatively wide, the magnitude of the point estimates provides a basis for future validation.

The identified SNPs mapped to genes which encode key m6A-related proteins, including the demethylase ALKBH5; methyltransferase complex components METTL3, WTAP, and ZC3H13; and the “reader” proteins YTHDC2 and YTHDF2. The dysregulation of m6A modifiers is increasingly recognized as a common driver of drug resistance in various cancers.

For *METTL3*, two variants (rs1263793 and rs1139130) were associated with longer PFS, suggesting a potential protective effect. Existing studies have suggested that long-term application of IM may activate m6A methyltransferase METTL3, thereby increasing the m6A modification level of the multidrug transporter MRP1 and promoting its translation, ultimately driving IM resistance in GIST [13]. The two SNPs we identified within *METTL3* may contribute to resistance if they alter METTL3 function or expression, though this remains speculative. *METTL3* rs1139130 has been previously associated with multiple disease phenotypes in independent populations, suggesting it may be a functionally relevant variant [20,21,22]. Although those studies involved different disease contexts and were also observational, the recurrent associations at this locus across studies suggest it may represent a functionally relevant variant. The rs1139130 was predicted to affect exonic splicing function, using the online software SNPinfo (National Institute of Environmental Health Sciences, NIH, Research Triangle Park, NC, USA; https://snpinfo.niehs.nih.gov/snpinfo/snpfunc.html, accessed on 10 February 2026), providing biological plausibility for a potential impact on METTL3 activity. Collectively, these observations support further investigation of rs1139130 in functional validation and independent replication studies.

For WTAP and ZC3H13, functional evidence in GIST is currently lacking. Given their shared role in the m6A methyltransferase complex, it is plausible that WTAP and ZC3H13 may contribute to imatinib resistance through mechanisms analogous to those proposed for METTL3. Of note, the *WTAP* rs2758313 identified in our study is in linkage disequilibrium with another variant, rs1853259, located in a predicted enhancer region, which has been associated with clinical outcomes in several independent studies [20,23]. While this does not constitute direct evidence for a functional role of the genotyped SNP, it suggests that the observed association may tag a regulatory variant in this region.

Among the m6A pathway genes, *ALKBH5* rs71372273 showed a strong association with PFS in our cohort. To date, no studies have directly investigated the role of ALKBH5 in imatinib resistance in GIST. However, given that imatinib is a tyrosine kinase inhibitor (TKI), insights may be gleaned from studies of other TKI resistance in different cancer types. In EGFR-TKI-resistant non-small cell lung cancer, ALKBH5 downregulation contributes to TKI resistance via m6A-mediated immunosuppression [24]. While the cancer context differs from our study, this finding suggests that ALKBH5-mediated m6A demethylation may represent a mechanism of TKI resistance worthy of investigation in imatinib-treated GIST. However, considering that the CI of this result is wide and the sample size for the subgroup is small, the interpretation should be performed with caution.

Additionally, no studies have investigated the role of YTHDC2 or YTHDF2 in IM resistance so far. YTHDC2 and YTHDF2 can bind to m6A-modified sites and reduce the stability of target mRNAs [25,26]. Evidence from other cancer types suggests that these m6A readers may contribute to TKI resistance, raising the possibility that they could also be relevant in IM resistance [27,28]. These findings, though indirect, support further investigation of YTHDC2 and YTHDF2 in the context of imatinib resistance in GIST. The association of multiple polymorphisms in m6A regulatory genes with PFS in this study suggests that the m6A regulatory network may contribute to inter-patient variability in imatinib response. These findings provide a foundation for future functional studies and independent validation. For several identified SNPs located in non-coding regions, no functional predictions were available. Their potential relevance cannot be excluded and requires future studies to elucidate their biological role.

To further explore the cumulative effect of genetic variations within this pathway, we constructed a genetic risk score based on the identified m6A-related SNPs. Compared to the low-risk group, both the intermediate-risk group (HR = 3.40, 95% CI: 1.15–10.07, *p* = 0.027) and the high-risk group (HR = 9.42, 95% CI: 2.57–34.44, *p* < 0.001) were associated with shorter PFS even after multivariable adjustment for demographic and clinical factors. The wide confidence intervals for both the intermediate- and high-risk groups were likely because of the limited number of events in the low-risk reference group, suggesting the need for validation in larger cohorts with more events across all risk levels. Additionally, the GRS showed a graded association with PFS (HR per grade increase = 3.05, 95% CI: 1.62–5.75, *p* for trend < 0.001), suggesting that patients in our cohort carrying a higher burden of risk alleles had progressively shorter PFS. These findings suggest that the cumulative burden of risk variants across multiple genes within the m6A pathway may collectively influence the m6A regulatory network, potentially contributing to inter-patient variability in imatinib response. Although the GRS stratified patients in this cohort, the score does not support immediate clinical implementation and should currently be regarded as a research tool requiring prospective validation in independent cohorts. Future studies may explore whether integrating pathway-level genetic information could refine risk stratification and inform therapeutic strategies.

*ATG5* encodes a critical component in autophagosome formation, and the SNP rs117827198 within it was associated with shorter PFS (HR = 10.49, 95% CI: 2.47–44.59, *p* = 0.001). The HR is considerably large, with a value over 10, though the wide confidence interval is likely due to the small sample size in the subgroup. The findings need to be consistently framed as hypothesis-generating observations that require validation. Gao et al. reported that IM preferentially induces the deubiquitinase USP13 in GIST cells, which stabilizes ATG5 through a PAK1-dependent deubiquitination process, thereby enhancing autophagy and promoting IM resistance [10]. This provides biological plausibility for the association observed in our study. ATG5-mediated autophagy has also been implicated in resistance to EGFR-TKIs in lung adenocarcinoma cells, suggesting that autophagy may represent a common mechanism of TKI resistance across tumor types [29]. To our knowledge, this is the first report of an association between an ATG5 polymorphism and clinical outcomes in imatinib-treated GIST patients. These findings need further investigation in independent cohorts and functional studies.

Our analysis also identified SNPs in two DNA damage repair genes, *MBD4* rs140696 (HR = 3.84, 95% CI: 1.07–13.81, *p* = 0.040) and *ERCC6* rs4253231 (HR = 4.83, 95% CI: 1.16–20.03, *p* = 0.030), that were associated with shorter PFS in our patient cohort. The wide confidence intervals reflect the small number of variant carriers for both SNPs and indicate that these findings require confirmation in larger cohorts. MBD4 functions as a thymine DNA glycosylase in base excision repair [30]. In IM-resistant chronic myelogenous leukemia cells, lower levels of DNA damage and higher MBD4 expression have been observed compared to sensitive cells [31,32]. The study provided biological plausibility for a potential role of DNA repair capacity in modulating imatinib response. ERCC6 is a key component of the nucleotide excision repair pathway [33]. *ERCC6* rs4253231 is predicted to be microRNA-binding sites, suggesting a potential post-transcriptional regulatory mechanism that warrants further investigation. Although DNA damage repair pathways have been firmly linked to drug resistance in multiple cancer types, the specific roles of MBD4 and ERCC6 in GISTs have not yet been systematically characterized. Our findings suggest that polymorphisms in DNA repair-related genes may be associated with clinical outcomes in imatinib-treated GIST patients, but the small number of variant carriers and the exploratory nature of this analysis necessitate validation in larger cohorts and further functional investigation.

In this study, we evaluated the associations between genetic polymorphisms and PFS after adjusting for clinical covariates. In our cohort, no significant association was found for tumor location or maximum tumor diameter with PFS, which differs from some previous reports [34,35]. Given that our cohort consisted of patients with an initial response to IM, the relationship between these baseline parameters and the timing of secondary resistance may be less direct. However, a high mitotic index (>5/50 HPF) was associated with shorter PFS, consistent with previous studies [36].

Although multivariable Cox regression and risk score matching were employed, residual confounding from unmeasured factors cannot be excluded. Key prognostic variables, including performance status, frequency and sensitivity of radiographic assessments, and supportive care, were not systematically captured and may be unevenly distributed across genetic subgroups, potentially influencing the observed associations. As a single-center study, the relative consistency in healthcare access and treatment protocols within our cohort may reduce confounding from institutional variability. However, this also limits the generalizability of our findings. Additionally, data on key molecular tumor characteristics (e.g., KIT/PDGFRA mutation subtypes) were available only for a subset of patients, precluding their adjustment in the primary multivariable model. Therefore, the observed associations should be interpreted as preliminary, hypothesis-generating evidence, not causation. This associative hypothesis requires validation in prospective and multi-center studies with stricter control and measurement of the aforementioned clinical and molecular confounders.

Overall, our study identified 12 SNPs in genes involved in m6A modification, autophagy, and DNA damage repair that were associated with PFS in our cohort of imatinib-treated GIST patients. Ten of the SNPs remained associated after adjustment for standard clinical factors. The cumulative effect of m6A pathway variants further suggests that pathway-level genetic assessment may capture aspects of inter-patient variability not explained by individual SNPs alone. To our knowledge, this is the first systematic evidence that germline polymorphisms in m6A modification, autophagy, and DNA damage repair pathways are associated with clinical outcomes in imatinib-treated GIST patients. However, the identified SNPs and the risk score approach are not ready for clinical application. These findings provide a foundation for future studies aimed at validating the role of these variants in independent cohorts and exploring their potential biological mechanisms.

In summary, the main limitations of this study are as follows. First, the most significant limitation of this study is its single-center, retrospective, observational design. The single-center retrospective design may introduce bias and limit generalizability, and residual confounding from unmeasured clinical factors cannot be excluded. Second, in this exploratory study, *p* values were reported without adjustment for multiple comparisons. The findings should therefore be regarded as preliminary results requiring validation in independent cohorts. Third, the modest sample size, particularly for rare genotype subgroups, resulted in imprecise estimates and limited our ability to perform robust stratified analyses. Fourth, the candidate-gene approach may have missed relevant variants in other pathways. Finally, given the hypothesis-generating nature of this study, the mechanistic roles of the identified SNPs remain speculative and require functional validation.

Future studies should prioritize validation of these findings in larger, independent, and ideally prospective cohorts with a comprehensive collection of clinical and molecular covariates. Functional investigations are also needed to elucidate the biological basis of the observed associations. If validated, these genetic biomarkers could eventually inform a more refined approach to risk stratification in GIST, though they are not yet ready for clinical implementation.

## 5. Conclusions

In this study, we identified multiple SNPs in genes related to m6A modification, autophagy, and DNA damage repair that were associated with disease progression after adjustment for established clinical characteristics. A cumulative risk score based on m6A pathway variants demonstrated the ability to stratify patients into distinct prognostic groups, suggesting that pathway-level genetic assessment may serve as a prognostic research tool.

These findings provide preliminary evidence that genetic variation may contribute to inter-patient differences in clinical outcomes among IM-treated GIST patients. The proposed genetic risk score is not yet ready for clinical implementation and should currently be regarded as a promising research tool requiring prospective validation. Although further validation in larger, independent cohorts is essential, our results offer hypothesis-generating support for the prognostic relevance of genetic polymorphisms in patients receiving IM and suggest that incorporating pathway-level genetic information could be further explored in risk stratification for disease progression in IM-treated GIST.

## Figures and Tables

**Table 1:** Baseline patient characteristics and univariate association with PFS.

Character		Events, *n* (%)	HR (95%CI)	*p* Value
Age, years				
Median (range)	55 (22–79)	39 (100%)	0.99 (0.96–1.01)	0.38
BSA, m^2^				
Median (range)	1.71 (1.36–2.11)	39 (100%)	5.76 (0.80–41.56)	0.082
Sex, *n* (%)				
Female	79 (45.9%)	17 (43.6%)	1.00 (Reference)	
Male	93 (54.1%)	22 (56.4%)	0.90 (0.48–1.69)	0.737
Tumor site, *n* (%)				
Stomach	110 (64.0%)	19 (48.7%)	1.00 (Reference)	
Small intestine	44 (25.6%)	16 (41.0%)	1.94 (1.00–3.79)	0.051
Other	18 (10.5%)	4 (10.3%)	1.09 (0.37–3.21)	0.872
Tumor maximum diameter, *n* (%)				
≤2 cm	7 (4.1%)	2 (5.1%)	1.00 (Reference)	
2–5 cm	29 (16.9%)	4 (10.3%)	0.55 (0.10–3.03)	0.495
5–10 cm	97 (56.4%)	21 (53.8%)	0.89 (0.21–3.84)	0.879
≥10 cm	39 (22.7%)	12 (30.8%)	1.46 (0.32–6.55)	0.624
per one-category increase			1.39 (0.89–2.18)	0.146
Mitotic index, *n* (%)				
≤5/50 HPF	71 (41.3%)	9 (23.1%)	1.00 (Reference)	
>5/50 HPF	87 (50.6%)	28 (71.8%)	2.53 (1.19–5.38)	0.016
Untested	14 (8.1%)	2 (5.1%)	1.98 (0.43–9.23)	0.383
Tumor somatic mutation, *n* (%)				
KIT exon 11	93 (54.1%)	17 (43.6%)	1.00 (Reference)	
KIT exon 9	7 (4.1%)	3 (7.7%)	2.18 (0.64–7.46)	0.215
KIT exon 17	1 (0.6%)	1 (2.6%)	7.65 (1.01–58.51)	0.05
Wild type	20 (11.6%)	5 (12.8%)	1.34 (0.49–3.63)	0.568
Untested	51 (29.7%)	13 (33.3%)	1.43 (0.69–2.95)	0.34

PFS: progression-free survival; HR: hazard ratio; CI: confidence interval; BSA: body surface area; HPF: high-power fields.

**Table 2:** Univariate association of candidate SNPs with PFS in the 6-month landmark cohort.

Pathway	Gene Variant	Patient, *n* (%)	Events, *n*	HR (95%CI)	*p* Value
M6A modification					
	*ALKBH5* rs71372273	171	39		
	TT	146 (85.4%)	31 (79.5%)	1.00 (Reference)	
	TA	23 (13.5%)	6 (15.4%)	1.25 (0.52–3.01)	0.615
	AA	2 (1.2%)	2 (5.1%)	102.87 (14.11–749.77)	<0.001
	per one-category increase			2.16 (1.06–4.37)	0.033
	TT + TA	169 (98.8%)	37 (94.9%)	1.00 (Reference)	
	AA	2 (1.2%)	2 (5.1%)	99.49 (13.72–721.52)	<0.001
	* *METTL3* rs1139130	150	30		
	AA	55 (36.7%)	15 (50.0%)	1.00 (Reference)	
	AG	70 (46.7%)	11 (36.7%)	0.48 (0.22–1.05)	0.067
	GG	25 (16.7%)	4 (13.3%)	0.43 (0.14–1.33)	0.143
	per one-category increase			0.60 (0.34–1.03)	0.064
	AA	55 (36.7%)	15 (50.0%)	1.00 (Reference)	
	AG + GG	95 (63.3%)	15 (50.0%)	0.47 (0.23–0.97)	0.035
	*METTL3* rs1263793	172	39		
	AA	60 (34.9%)	18 (46.2%)	1.00 (Reference)	
	AG	80 (46.5%)	16 (41.0%)	0.59 (0.30–1.16)	0.123
	GG	32 (18.6%)	5 (12.8%)	0.41 (0.15–1.10)	0.078
	per one-category increase			0.62 (0.39–0.99)	0.046
	AA	60 (34.9%)	18 (46.2%)	1.00 (Reference)	
	AG + GG	112 (65.1%)	21 (53.8%)	0.53 (0.28–1.00)	0.047
	*WTAP* rs2758313	172	39		
	CC	62 (36.0%)	9 (23.1%)	1.00 (Reference)	
	CG	87 (50.6%)	23 (59.0%)	1.53 (0.76–3.06)	0.231
	GG	23 (13.4%)	7 (17.9%)	3.14 (1.29–7.64)	0.012
	per one-category increase			1.61 (1.02–2.53)	0.041
	CC	62 (36.0%)	9 (23.1%)	1.00 (Reference)	
	CG + GG	110 (64.0&)	30 (76.9%)	2.19 (1.04–4.61)	0.035
	*YTHDC2* rs114925657	172	39		
	TT	139 (80.8%)	34 (87.2%)	1.00 (Reference)	
	TC	32 (18.6%)	4 (10.3%)	0.42 (0.15–1.18)	0.101
	CC	1 (0.6%)	1 (2.6%)	50.29 (5.22–484.37)	<0.001
	per one-category increase			0.65 (0.27–1.56)	0.334
	TT + TC	171 (99.4%)	38 (97.4%)	1.00 (Reference)	
	CC	1 (0.6%)	1 (2.6%)	56.50 (5.88–543.15)	<0.001
	*YTHDC2* rs1833678	171	38		
	TT	150 (87.7%)	29 (76.3%)	1.00 (Reference)	
	TC	20 (11.7%)	9 (23.7%)	2.44 (1.15–5.19)	0.020
	CC	1 (0.6%)	0 (0%)	-	-
	per one-category increase			1.82 (0.94–3.53)	0.077
	TT	150 (87.7%)	29 (76.3%)	1.00 (Reference)	
	TC + CC	21 (12.3%)	9 (23.7%)	2.26 (1.06–4.81)	0.030
	*YTHDF2* rs7520756	172	39		
	CC	97 (56.4%)	16 (41.0%)	1.00 (Reference)	
	CT	61 (35.5%)	16 (41.0%)	1.53 (0.76–3.06)	0.231
	TT	14 (8.1%)	7 (17.9%)	3.14 (1.29–7.64)	0.012
	per one-category increase			1.72 (1.11–2.68)	0.016
	CC + CT	158 (91.9%)	32 (82.1%)	1.00 (Reference)	
	TT	14 (8.1%)	7 (17.9%)	2.59 (1.14–5.89)	0.018
	*ZC3H13* rs17703559	172	39		
	AA	115 (66.9%)	31 (79.5%)	1.00 (Reference)	
	AG	51 (29.7%)	7 (17.9%)	0.49 (0.22–1.12)	0.091
	GG	6 (3.5%)	1 (2.6%)	0.32 (0.04–2.35)	0.261
	per one-category increase			0.52 (0.27–1.01)	0.053
	AA	115 (66.9%)	31 (79.5%)	1.00 (Reference)	
	AG + GG	57 (33.1%)	8 (20.5%)	0.46 (0.21–1.00)	0.045
	*ZC3H13* rs7318648	172	39		
	TT	98 (57.0%)	17 (43.6%)	1.00 (Reference)	
	TC	61 (35.5%)	20 (51.3%)	2.22 (1.16–4.25)	0.016
	CC	13 (7.6%)	2 (5.1%)	1.01 (0.23–4.38)	0.991
	per one-category increase			1.42 (0.90–2.25)	0.132
	TT	98 (57.0%)	17 (43.6%)	1.00 (Reference)	
	TC + CC	74 (43.0%)	22 (56.4%)	2.00 (1.06–3.78)	0.029
Autophagy					
	* *ATG5* rs117827198	145	30		
	CC	136 (93.8%)	26 (86.7%)	1.00 (Reference)	
	CT	8 (5.5%)	4 (13.3%)	7.77 (2.54–23.78)	<0.001
	TT	1 (0.7%)	0 (0%)	-	-
	per one-category increase			2.78 (1.21–6.36)	0.016
	CC	136 (93.8%)	26 (86.7%)	1.00 (Reference)	
	CT + TT	9 (6.2%)	4 (13.3%)	5.98 (1.98–18.05)	<0.001
DNA damage repair					
	*ERCC6* rs4253231	171	39		
	AA	126 (73.7%)	29 (74.4%)	1.00 (Reference)	
	AG	40 (23.4%)	7 (17.9%)	0.66 (0.29–1.50)	0.322
	GG	5 (2.9%)	3 (7.7%)	3.51 (1.05–11.70)	0.041
	per one-category increase			1.11 (0.61–2.03)	0.728
	AA + AG	166 (97.1%)	36 (92.3%)	1.00 (Reference)	
	GG	5 (2.9%)	3 (7.7%)	3.85 (1.17–12.72)	0.017
	*MBD4* rs140696	171	39		
	GG	129 (75.4%)	29 (74.4%)	1.00 (Reference)	
	GA	38 (22.2%)	7 (17.9%)	0.89 (0.39–2.03)	0.777
	AA	4 (2.3%)	3 (7.7%)	4.34 (1.31–14.39)	0.016
	per one-category increase			1.40 (0.78–2.52)	0.257
	GG + GA	167 (97.7%)	36 (92.3%)	1.00 (Reference)	
	AA	4 (2.3%)	3 (7.7%)	4.45 (1.36–14.59)	0.007

* The analysis for rs1139130 and rs117827198 was based on the subset of patients with available genotype data. SNP: single- nucleotide polymorphism.

**Table 3:** Multivariable-adjusted association of individual SNPs with PFS in the 6-month landmark cohort.

Pathway	Gene Variant	Comparison vs. Reference	* Adjusted HR (95%CI)	Adjusted *p* Value
M6A modification				
	*ALKBH5* rs71372273	AA vs. TT + TA	36.40 (4.70–281.67)	<0.001
	*METTL3* rs1139130	AG + GG vs. AA	0.46 (0.21–0.99)	0.049
	*METTL3* rs1263793	AG + GG vs. AA	0.40 (0.20–0.83)	0.014
	*WTAP* rs2758313	CG + GG vs. CC	2.05 (0.92–4.57)	0.079
	*YTHDC2* rs114925657	CC vs. TT + TC	13.70 (1.27–147.79)	0.031
	*YTHDC2* rs1833678	TC + CC vs. TT	2.87 (1.18–7.03)	0.021
	*YTHDF2* rs7520756	TT vs. CC + CT	2.34 (0.96–5.74)	0.062
	*ZC3H13* rs17703559	AG + GG vs. AA	0.44 (0.20–0.99)	0.049
	*ZC3H13* rs7318648	TC + CC vs. TT	2.31 (1.15–4.61)	0.018
Autophagy				
	*ATG5* rs117827198	CT + TT vs. CC	10.49 (2.47–44.59)	0.001
DNA damage repair				
	*ERCC6* rs4253231	GG vs. AA + AG	4.83 (1.16–20.03)	0.030
	*MBD4* rs140696	AA vs. GG + GA	3.84 (1.07–13.81)	0.040

* Adjusted for age, sex, BSA, tumor location, maximum diameter and mitotic index.

**Table 4:** Cox proportional hazards analysis of PFS according to the GRS based on m6A pathway SNPs in the 6-month landmark cohort.

Risk Group	Patient, *n* (%)	Events, *n* (%)	HR (95% CI)	*p* Value	* Adjusted HR (95% CI)	Adjusted *p* Value
Total	149	30				
Low-risk	65 (43.6%)	5 (16.7%)	1.00 (Reference)		1.00 (Reference)	
Intermediate-risk	68 (45.6%)	16 (53.3%)	3.85 (1.41–10.56)	0.009	3.40 (1.15–10.07)	0.027
High-risk	16 (10.7%)	9 (30.0%)	9.56 (3.19–28.67)	<0.001	9.42 (2.57–34.44)	<0.001
per one-category increase			3.00 (1.80–4.98)	<0.001	3.05 (1.62–5.75)	<0.001

* Adjusted for age, sex, BSA, tumor location, maximum diameter and mitotic index. GRS: genetic risk score.

## Data Availability

Compiled data is reported in the tables above. The raw data files are available from the authors upon request.

## Supplementary Materials

The following supporting information can be downloaded at https://www.mdpi.com/article/10.3390/jcm15062183/s1, Table S1: List of candidate genes in IM resistance-related pathways; Table S2: Hardy–Weinberg equilibrium test of SNPs.

## Abbreviations

The following abbreviations are used in this manuscript:

IM	imatinib mesylate
GIST	gastrointestinal stromal tumors
SNP	single-nucleotide polymorphism
PFS	progression-free survival
NCCN	National Comprehensive Cancer Network
BSA	body surface area
MALDI-TOF	matrix-assisted laser desorption/ionization time-of-flight
HWE	Hardy–Weinberg equilibrium
HR	hazard ratio
CI	confidence interval
HPF	high-power fields
GRS	genetic risk score
TKI	tyrosine kinase inhibitor
