# Supplementary material for: Genetic Variations in the M6A Modification Pathway as Potential Predictors of Imatinib Secondary Resistance in Gastrointestinal Stromal Tumors
Source: J Clin Med. 2026 Mar 13;15(6):2183. doi: 10.3390/jcm15062183 (PMC13027091; doi:10.3390/jcm15062183)
Supplement: Supplementary file 1 [file jcm-15-02183-s001.zip › jcm-4115107-supplementary.pdf]

Supplementary Table S1. List of candidate genes in Imatinib mesylate resistance-related pathways.

Pathway	Gene	Reference
**M6A modification**		
	ALKBH5	Yu F, et al. 2025[1]; Hong L, et al. 2023[2]; Zhang W, et al. 2025[3]
	FTO	Lin K, et al. 2023[4]; Lin Z, et al. 2023[5]
	HNRNPC	Pan Y, et al. 2023[6]; Yang G, et al. 2025[7]
	METTL14	Zhang J, et al. 2025[8]; Ianniello Z, et al.2021[9]; Chen Y, et al. 2022[10]
	METTL3	Gao Z, et al. 2023[11]; Xu K, et al. 2022[12]; Ianniello Z, et al. 2021[9]
	RBM15	Li H, et al. 2025[13]; Hou M, et al. 2020[14]
	VIRMA	Miranda-Gonçalves V, et al. 2021[15]
	WTAP	Hong L, et al. 2023[2]; Liu Y, et al. 2023[16]
	YTHDC1	Li W, et al. 2022[17]
	YTHDC2	Wang X, et al. 2023[18]; Chen L, et al. 2023[19]
	YTHDF1	Xu K, et al. 2022[12]
	YTHDF2	Liao Y, et al. 2023[20]; Jiang J, et al. 2022[21]
	ZC3H13	Lin X, et al. 2022[22]
**DNA damage repair**		
	ALKBH2	Johannessen TC, et al. 2013[23]; Li J, et al. 2025[24]; Gao W, et al. 2011[25]
	ALKBH3	Gutierrez R, et al. 2024[26]; Li J, et al. 2025[24]; Knijnenburg TA, et al. 2018[27]
	APEX1	Assämäki R, et al. 2007[28]; Zhan Z, et al. 2024[29]; He H, et al. 2021[30]
	ATR	Shi E, et al. 2016[31]; Cote GM, et al. 2025[32]; Morii M, et al. 2015[33]
	BARD1	Tarsounas M, et al. 2020[34]; Zhang L, et al. 2025[35]; Tai Y, et al. 2024[36]
	BLM	Ophir G, et al. 2021[37]; Wojnicki K, et al. 2025[38]; Slupianek A, et al. 2005[39]
	BRCA1	Ju DS, et al. 2007[40]; Feng Y, et al. 2021[41]
	BRCA2	Feng Y, et al. 2021[41]; Cui S, et al. 2025[42]; Waisbren J, et al. 2015[43]
	CHEK2	Denu RA, et al. 2024[44]; Casetti L, et al. 2013[45]; Nishida T, et al. 2024[46]
	EIF4A2	van der Wijngaart H, et al. 2023[47]; Chen ZH, et al. 2019[48]; Liu M, et al. 2019[49]
	ERCC6	Zhao Z, et al. 2017[50]; Chen C, et al. 2023[51]; Xu R, et al. 2025[52]
	EXO1	Qi L, et al. 2019[53]; O'Sullivan J, et al. 2023[54]; He D, et al. 2020[55]

	EXO5	Knijnenburg TA, et al. 2018[27]; Mazza F, et al. 2025[56]
	FANCM	Liu Z, et al. 2024[57]; Zhang Y, et al. 2022[58]
	LIG3	Kifayat K, et al. 2025[59]; Paes Dias M, et al. 2021[60]; Ali R, et al. 2021[61]
	MBD4	Bader SA, et al. 2007[62]; Dinis J, et al. 2012[63]
	MGMT	Li J, et al. 2025[24]; Knijnenburg TA, et al. 2018[27]
	MSH2	Feng Y, et al. 2021[41]; Bara T, et al. 2015[64]; Li B, et al. 2020[65]
	NEIL2	Anurag M, et al. 2018[66]; Kadioglu O, et al. 2021[67]; He W, et al.2020[68]
	NEIL3	Lai HH, et al. 2022[69]; Wang Y, et al. 2021[70]
	PARP1	Orlikova-Boyer B, et al. 2024 [71]; Xin J, et al. 2023[72]; Kulkarni S, et al. 2025[73]
	PARP2	Assämäki R, et al. 2007[28]; Kulkarni S, et al. 2025[73]
	PARP4	Sung HY, et al. 2023[74]; Kozono D, et al. 2024[75]
	PMS2	Sever T, et al. 2012[76]; Li B, et al. 2020[65]; Fu Y, et al. 2023[77]
	POLD1	Anurag M, et al. 2022[78]
	POLE	Feng Y, et al. 2021[41]G
	POLM	Xiao Y, et al. 2024[79]
	POLQ	Kulkarni S, et al. 2025[73]
	PRKDC	Zhang W, et al. 2024[80]; Zhao P, et al. 2023[81]
	RECQL	Zhang P, et al. 2006[82]; Viziteu E, et al. 2017[83]
	RIF1	Harvey-Jones E, et al. 2024[84]
	SLX4	Guidi L, et al. 2023[85]; Engel JL, et al. 2024[86]; Zhao X, et al. 2025[87]
	TP53	Cao J, et al. 2018[88]; Song B, et al. 2024[89]; Li B, et al. 2020[65]
	TP53BP1	Mirza-Aghazadeh-Attari M, et al. 2019[90]; Schouten PC, et al. 2016[91]; Harvey-Jones E, et al. 2024[84]
	WRN	Cheng WH, et al. 2003[92]; Picco G, et al. 2021[93]
	XRCC4	Mi L, et al. 2025[94]; Xu M, et al. 2022[95]
**Autophagy**		
	ATG12	Zhang R, et al. 2024[96]; Dai H, et al. 2021[97]; Chen Y, et al. 2021[98]
	ATG5	Gao Z, et al. 2023[11]; Yu Y, et al. 2012[99]; Yue P, et al. 2025[100]

BECN1	Chen W, et al. 2020[101]; Zhang J, et al. 2022[102]
ULK1	Cao HX, et al. 2020[103]; Han SH, et al. 2019[104]
XIAP	Xie Q, et al. 2017[105]; Roy R, et al. 2025[106]

Supplementary Table S2. List of selected tag SNPs and Hardy-Weinberg equilibrium test results.

Pathway	Gene	SNP	*p* Value	Gene	SNP	*p* Value
**M6A modification**						
	ALKBH5	rs118025073	NS	RBM15	rs3738751	NS
		rs2124370	NS		rs3738752	NS
		rs58804619	NS		rs4839342	**< 0.0001**
		rs71372273	NS		rs4839343	NS
		rs75364952	NS		rs814771	NS
		rs9913266	NS	VIRMA	rs12677404	NS
	FTO	rs12599807	**< 0.0001**		rs2381831	NS
		rs2192869	NS		rs4734279	NS
		rs2192870	NS		rs75537904	NS
		rs2192871	**< 0.0001**		rs76318016	NS
		rs2192872	NS	WTAP	rs11752345	**< 0.0001**
		rs2689251	NS		rs1322429	**< 0.0001**
		rs3764307	NS		rs2758313	NS
		rs4783818	NS	YTHDC1	rs57619473	**0.0064**
		rs58340747	NS		rs62316045	**0.0032**
		rs856981	NS		rs79454882	NS
		rs856982	NS	YTHDC2	rs114925657	NS
		rs856983	NS		rs117018259	NS
	HNRNPC	rs12878905	NS		rs139865708	NS
		rs34514090	**< 0.0001**		rs1816062	NS
		rs7147840	NS		rs1833678	NS
		rs72675012	NS		rs2910018	NS
		rs78974307	NS		rs2914150	NS
		rs8016099	NS		rs73242858	NS
	METTL14	rs10213474	NS		rs78284628	NS
		rs1064034	NS		rs79242542	**0.0014**
		rs115267066	**< 0.0001**	YTHDF1	rs13042920	NS
		rs17050450	NS		rs6090296	**< 0.0001**
		rs4834698	NS	YTHDF2	rs12135002	**0.036**
	METTL3	rs10450908	NS		rs74640552	NS
		rs1061026	NS		rs7520756	NS
		rs1139130	NS		rs79215510	NS
		rs114236505	NS	ZC3H13	rs17067523	NS
		rs1263793	NS		rs17703559	NS

	rs1263800	NS		rs4942469	NS
	rs1263801	**0.025**		rs7317990	NS
	rs1268403	NS		rs7318648	NS
	rs17197156	NS		rs7328081	NS
	rs1794219	NS		rs75590836	NS
	rs4417466	NS		rs79315218	NS
				rs9534290	**0.011**
**DNA damage repair**					
ALKBH2	rs76283368	NS	NEIL2	rs1534862	NS
ALKBH3	rs1130290	NS	NEIL3	rs13112358	NS
APEX1	rs1130409	NS	PARP1	rs1805404	NS
ATR	rs2227928	**0.0011**		rs1805405	**0.045**
BARD1	rs16852600	NS	PARP2	rs2297616	NS
	rs2229571	**< 0.0001**	PARP4	rs1050110	NS
BLM	rs3815003	NS		rs7140044	NS
BRCA1	rs16941	NS	PMS2	rs1805321	**0.011**
	rs16942	NS	POLD1	rs1726801	**< 0.0001**
	rs1799966	NS		rs2445837	NS
	rs799917	NS	POLE	rs5744751	NS
BRCA2	rs144848	NS	POLM	rs3218660	NS
	rs1799944	NS		rs7778884	NS
CHEK2	rs16986640	NS	POLQ	rs2306211	NS
EIF4A2	rs1047148	NS	PRKDC	rs7830743	NS
ERCC6	rs2228527	NS	RECQL	rs10841834	NS
	rs4253211	NS		rs11046076	NS
	rs4253231	NS		rs2159943	NS
EXO1	rs1047840	NS	RIF1	rs117711862	NS
	rs4149963	NS		rs151107720	NS
	rs9350	NS		rs3732305	NS
EXO5	rs11208299	NS	SLX4	rs3810813	**< 0.0001**
FANCM	rs1367580	NS	TP53	rs2287497	**0.011**
	rs58790242	**< 0.0001**	TP53BP1	rs62316048	NS
	rs78211950	NS		rs689647	NS
LIG3	rs1052536	NS		rs690367	NS
MBD4	rs140696	NS	WRN	rs1346044	NS
MGMT	rs12917	NS	XRCC4	rs3734091	**0.024**
MSH2	rs7566192	**< 0.0001**			
**Autophagy**					
ATG5	rs117827198	NS	ULK1	rs1134574	NS
	rs12212740	NS			

Reference

1. Yu, F.; Zheng, S.; Yu, C.; Gao, S.; Shen, Z.; Nar, R.; Liu, Z.; Huang, S.; Wu, L.; Gu, T.; et al.

KRAS mutants confer platinum resistance by regulating ALKBH5 posttranslational modifications in lung cancer. *The Journal of clinical investigation* **2025**, *135*, e185149, doi:10.1172/jci185149.
2. Hong, L.; Wang, X.; Zheng, L.; Wang, S.; Zhu, G. Tumor-associated macrophages promote cisplatin resistance in ovarian cancer cells by enhancing WTAP-mediated N6-methyladenosine RNA methylation via the CXCL16/CXCR6 axis. *Cancer chemotherapy and pharmacology* **2023**, *92*, 71-81, doi:10.1007/s00280-023-04533-8.
3. Zhang, W.; Wang, J.; Liang, J.; He, Z.; Wang, K.; Lin, H. RNA methylation of CD47 mediates tumor immunosuppression in EGFR-TKI resistant NSCLC. *British journal of cancer* **2025**, *132*, 569-579, doi:10.1038/s41416-025-02945-2.
4. Lin, K.; Zhou, E.; Shi, T.; Zhang, S.; Zhang, J.; Zheng, Z.; Pan, Y.; Gao, W.; Yu, Y. m6A eraser FTO impairs gemcitabine resistance in pancreatic cancer through influencing NEDD4 mRNA stability by regulating the PTEN/PI3K/AKT pathway. *Journal of experimental & clinical cancer research : CR* **2023**, *42*, 217, doi:10.1186/s13046-023-02792-0.
5. Lin, Z.; Wan, A.H.; Sun, L.; Liang, H.; Niu, Y.; Deng, Y.; Yan, S.; Wang, Q.P.; Bu, X.; Zhang, X.; et al. N6-methyladenosine demethylase FTO enhances chemo-resistance in colorectal cancer through SIVA1-mediated apoptosis. *Molecular therapy : the journal of the American Society of Gene Therapy* **2023**, *31*, 517-534, doi:10.1016/j.ymthe.2022.10.012.
6. Pan, Y.; Lu, X.; Shu, G.; Cen, J.; Lu, J.; Zhou, M.; Huang, K.; Dong, J.; Li, J.; Lin, H.; et al. Extracellular Vesicle-Mediated Transfer of LncRNA IGFL2-AS1 Confers Sunitinib Resistance in Renal Cell Carcinoma. *Cancer research* **2023**, *83*, 103-116, doi:10.1158/0008-5472.Can-21-3432.
7. Yang, G.; Shen, L.; Cui, M.; Yang, J. Novel RNA-methylase HNRNPC promotes gastric cancer tumorigenesis by triggering the lactate-induced ferroptosis resistance. *Frontiers in immunology* **2025**, *16*, 1612935, doi:10.3389/fimmu.2025.1612935.
8. Zhang, J.; Liao, Z.H.; Xu, Y.M.; Li, S.Q.; Zhong, F.M.; Zhang, L.; Yao, F.Y.; Bai, Q.; Yao, L.H.; Huang, B.; et al. The role of METTL14 in the progression of chronic myeloid leukemia. *Hematology (Amsterdam, Netherlands)* **2025**, *30*, 2535819, doi:10.1080/16078454.2025.2535819.
9. Ianniello, Z.; Sorci, M.; Ceci Ginistrelli, L.; Iaiza, A.; Marchioni, M.; Tito, C.; Capuano, E.; Masciarelli, S.; Ottone, T.; Attrotto, C.; et al. New insight into the catalytic -dependent and -independent roles of METTL3 in sustaining aberrant translation in chronic myeloid leukemia. *Cell death & disease* **2021**, *12*, 870, doi:10.1038/s41419-021-04169-7.
10. Chen, Y.; Lu, Z.; Qi, C.; Yu, C.; Li, Y.; Huan, W.; Wang, R.; Luo, W.; Shen, D.; Ding, L.; et al. N(6)-methyladenosine-modified TRAF1 promotes sunitinib resistance by regulating apoptosis and angiogenesis in a METTL14-dependent manner in renal cell carcinoma. *Molecular cancer* **2022**, *21*, 111, doi:10.1186/s12943-022-01549-1.
11. Gao, Z.; Li, C.; Sun, H.; Bian, Y.; Cui, Z.; Wang, N.; Wang, Z.; Yang, Y.; Liu, Z.; He, Z.; et al. N(6)-methyladenosine-modified USP13 induces pro-survival autophagy and imatinib resistance via regulating the stabilization of autophagy-related protein 5 in gastrointestinal stromal tumors. *Cell death and differentiation* **2023**, *30*, 544-559, doi:10.1038/s41418-022-01107-8.
12. Xu, K.; Zhang, Q.; Chen, M.; Li, B.; Wang, N.; Li, C.; Gao, Z.; Zhang, D.; Yang, L.; Xu, Z.; et al. N(6)-methyladenosine modification regulates imatinib resistance of gastrointestinal stromal tumor by enhancing the expression of multidrug transporter MRP1. *Cancer letters* **2022**, *530*,

85-99, doi:10.1016/j.canlet.2022.01.008.
13. Li, H.; Li, Y.; Zheng, X.; Chen, F.; Zhang, S.; Xu, S.; Mu, Y.; Shen, W.; Tong, J.; Chen, H.; et al. RBM15 facilitates osimertinib resistance of lung adenocarcinoma through m6A-dependent epigenetic silencing of SPOCK1. *Oncogene* **2025**, *44*, 307-321, doi:10.1038/s41388-024-03220-z.
14. Hou, M.; Guo, X.; Chen, Y.; Cong, L.; Pan, C. A Prognostic Molecular Signature of N^6-Methyladenosine Methylation Regulators for Soft-Tissue Sarcoma from The Cancer Genome Atlas Database. *Medical science monitor : international medical journal of experimental and clinical research* **2020**, *26*, e928400, doi:10.12659/msm.928400.
15. Miranda-Gonçalves, V.; Lobo, J.; Guimarães-Teixeira, C.; Barros-Silva, D.; Guimarães, R.; Cantante, M.; Braga, I.; Maurício, J.; Oing, C.; Honecker, F.; et al. The component of the m(6)A writer complex VIRMA is implicated in aggressive tumor phenotype, DNA damage response and cisplatin resistance in germ cell tumors. *Journal of experimental & clinical cancer research : CR* **2021**, *40*, 268, doi:10.1186/s13046-021-02072-9.
16. Liu, Y.; Da, M. Wilms tumor 1 associated protein promotes epithelial mesenchymal transition of gastric cancer cells by accelerating TGF-β and enhances chemoradiotherapy resistance. *Journal of cancer research and clinical oncology* **2023**, *149*, 3977-3988, doi:10.1007/s00432-022-04320-7.
17. Li, W.; Ye, K.; Li, X.; Liu, X.; Peng, M.; Chen, F.; Xiong, W.; Wang, Y.; Zhu, L. YTHDC1 is downregulated by the YY1/HDAC2 complex and controls the sensitivity of ccRCC to sunitinib by targeting the ANXA1-MAPK pathway. *Journal of experimental & clinical cancer research : CR* **2022**, *41*, 250, doi:10.1186/s13046-022-02460-9.
18. Wang, X.; Hu, Y.; Li, X.; Zhu, C.; Chen, F. YTHDC2-mediated m6A mRNA modification of Id3 suppresses cisplatin resistance in non-small cell lung cancer. *Journal of thoracic disease* **2023**, *15*, 1247-1257, doi:10.21037/jtd-23-187.
19. Chen, L.; Sun, K.; Qin, W.; Huang, B.; Wu, C.; Chen, J.; Lai, Q.; Wang, X.; Zhou, R.; Li, A.; et al. LIMK1 m(6)A-RNA methylation recognized by YTHDC2 induces 5-FU chemoresistance in colorectal cancer via endoplasmic reticulum stress and stress granule formation. *Cancer letters* **2023**, *576*, 216420, doi:10.1016/j.canlet.2023.216420.
20. Liao, Y.; Liu, Y.; Yu, C.; Lei, Q.; Cheng, J.; Kong, W.; Yu, Y.; Zhuang, X.; Sun, W.; Yin, S.; et al. HSP90β Impedes STUB1-Induced Ubiquitination of YTHDF2 to Drive Sorafenib Resistance in Hepatocellular Carcinoma. *Advanced science (Weinheim, Baden-Wurttemberg, Germany)* **2023**, *10*, e2302025, doi:10.1002/advs.202302025.
21. Jiang, J.; Fan, Q.; Qu, H.; Liu, C.; Liang, T.; Chen, L.; Huang, S.; Sun, X.; Chen, J.; Chen, T.; et al. Novel prognostic biomarkers, METTL14 and YTHDF2, associated with RNA methylation in Ewing's sarcoma. *Scientific reports* **2022**, *12*, 7041, doi:10.1038/s41598-022-06744-0.
22. Lin, X.; Wang, F.; Chen, J.; Liu, J.; Lin, Y.B.; Li, L.; Chen, C.B.; Xu, Q. N(6)-methyladenosine modification of CENPK mRNA by ZC3H13 promotes cervical cancer stemness and chemoresistance. *Military Medical Research* **2022**, *9*, 19, doi:10.1186/s40779-022-00378-z.
23. Johannessen, T.C.; Prestegarden, L.; Grudic, A.; Hegi, M.E.; Tysnes, B.B.; Bjerkvig, R. The DNA repair protein ALKBH2 mediates temozolomide resistance in human glioblastoma cells. *Neuro-oncology* **2013**, *15*, 269-278, doi:10.1093/neuonc/nos301.
24. Li, J.; Feng, X.; Liu, Z.; Deng, Y.; Sun, Z.; Chen, B.; Wu, L.; Wang, X.; Miao, L.; Zeng, L.; et al. USP7 promotes temozolomide resistance by stabilizing MGMT in glioblastoma. *Cell death*

& disease **2025**, *16*, 631, doi:10.1038/s41419-025-07969-3.

25. Gao, W.; Li, L.; Xu, P.; Fang, J.; Xiao, S.; Chen, S. Frequent down-regulation of hABH2 in gastric cancer and its involvement in growth of cancer cells. *Journal of gastroenterology and hepatology* **2011**, *26*, 577-584, doi:10.1111/j.1440-1746.2010.06531.x.
26. Gutierrez, R.; Chan, A.Y.S.; Lai, S.W.T.; Itoh, S.; Lee, D.H.; Sun, K.; Battad, A.; Chen, S.; O'Connor, T.R.; Shuck, S.C. Lack of mismatch repair enhances resistance to methylating agents for cells deficient in oxidative demethylation. *The Journal of biological chemistry* **2024**, *300*, 107492, doi:10.1016/j.jbc.2024.107492.
27. Knijnenburg, T.A.; Wang, L.; Zimmermann, M.T.; Chambwe, N.; Gao, G.F.; Cherniack, A.D.; Fan, H.; Shen, H.; Way, G.P.; Greene, C.S.; et al. Genomic and Molecular Landscape of DNA Damage Repair Deficiency across The Cancer Genome Atlas. *Cell reports* **2018**, *23*, 239-254, doi:10.1016/j.celrep.2018.03.076.
28. Assämäki, R.; Sarlomo-Rikala, M.; Lopez-Guerrero, J.A.; Lasota, J.; Andersson, L.C.; Llombart-Bosch, A.; Miettinen, M.; Knuutila, S. Array comparative genomic hybridization analysis of chromosomal imbalances and their target genes in gastrointestinal stromal tumors. *Genes, chromosomes & cancer* **2007**, *46*, 564-576, doi:10.1002/gcc.20439.
29. Zhan, Z.; Zhang, J.; Liang, H.; Wang, C.; Hong, L.; Liu, W. KAT6A Condensates Impair PARP1 Trapping of PARP Inhibitors in Ovarian Cancer. *Advanced science (Weinheim, Baden-Wurttemberg, Germany)* **2024**, *11*, e2400140, doi:10.1002/advs.202400140.
30. He, H.; Song, F.; Gao, Q.; Lu, Z.; Yuan, Y.; Li, X.; Chen, L.; Jia, C.; Yang, R.; Yang, J.; et al. The APEX1/miRNA-27a-5p axis plays key roles in progression, metastasis and targeted chemotherapy of gastric cancer. *International journal of pharmaceutics* **2021**, *599*, 120446, doi:10.1016/j.ijpharm.2021.120446.
31. Shi, E.; Chmielecki, J.; Tang, C.M.; Wang, K.; Heinrich, M.C.; Kang, G.; Corless, C.L.; Hong, D.; Fero, K.E.; Murphy, J.D.; et al. FGFR1 and NTRK3 actionable alterations in "Wild-Type" gastrointestinal stromal tumors. *Journal of translational medicine* **2016**, *14*, 339, doi:10.1186/s12967-016-1075-6.
32. Cote, G.M.; Kochupurakkal, B.S.; Do, K.; Bullock, A.; Cheng, M.L.; Muzikansky, A.; McLoughlin, D.E.; Cleary, J.M.; Gao, X.; Parikh, A.; et al. A Translational Study of the ATR Inhibitor Berzosertib as Monotherapy in Four Molecularly Defined Cohorts of Advanced Solid Tumors. *Clinical cancer research : an official journal of the American Association for Cancer Research* **2025**, *31*, 35-44, doi:10.1158/1078-0432.Ccr-24-1867.
33. Morii, M.; Fukumoto, Y.; Kubota, S.; Yamaguchi, N.; Nakayama, Y.; Yamaguchi, N. Imatinib inhibits inactivation of the ATM/ATR signaling pathway and recovery from adriamycin/doxorubicin-induced DNA damage checkpoint arrest. *Cell biology international* **2015**, *39*, 923-932, doi:10.1002/cbin.10460.
34. Tarsounas, M.; Sung, P. The antitumorigenic roles of BRCA1-BARD1 in DNA repair and replication. *Nature reviews. Molecular cell biology* **2020**, *21*, 284-299, doi:10.1038/s41580-020-0218-z.
35. Zhang, L.; Pan, Z.; Zhang, L.; Liu, H.; Li, Z.; Feng, S. Dysregulated BARD1 Contributes to Paclitaxel Resistance in Ovarian Cancer via Up-regulating CYP2C8. *Folia biologica* **2025**, *71*, 109-117, doi:10.14712/fb2025071030109.
36. Tai, Y.; Chow, A.; Han, S.; Coker, C.; Ma, W.; Gu, Y.; Estrada Navarro, V.; Kandpal, M.; Hibshoosh, H.; Kalinsky, K.; et al. FLT1 activation in cancer cells promotes PARP-inhibitor

resistance in breast cancer. *EMBO molecular medicine* **2024**, *16*, 1957-1980, doi:10.1038/s44321-024-00094-2.
37. Ophir, G.; Sivan, S.; Hana, S.; Guy, R.; Nathan, G.; Naomi, F.I.; Joseph, K.; Ido, W.; Ofer, M.; Yael, G.; et al. Abdominal Desmoid: Course, Severe Outcomes, and Unique Genetic Background in a Large Local Series. *Cancers* **2021**, *13*, 3673, doi:10.3390/cancers13153673.
38. Wojnicki, K.; Wojtas, B.; Ciechomska, I.A.; Kaza, B.; Guille, M.; Priebe, W.; Kaminska, B. Shared and non-overlapping functions of RECQL4 and BLM helicases in chemotherapeutics-induced glioma cell responses. *BMC cancer* **2025**, *25*, 1434, doi:10.1186/s12885-025-14932-0.
39. Slupianek, A.; Gurdek, E.; Koptyra, M.; Nowicki, M.O.; Siddiqui, K.M.; Groden, J.; Skorski, T. BLM helicase is activated in BCR/ABL leukemia cells to modulate responses to cisplatin. *Oncogene* **2005**, *24*, 3914-3922, doi:10.1038/sj.onc.1208545.
40. Ju, D.S.; Kim, M.J.; Bae, J.H.; Song, H.S.; Chung, B.S.; Lee, M.K.; Kang, C.D.; Lee, H.S.; Kim, D.W.; Kim, S.H. Camptothecin acts synergistically with imatinib and overcomes imatinib resistance through Bcr-Abl independence in human K562 cells. *Cancer letters* **2007**, *252*, 75-85, doi:10.1016/j.canlet.2006.12.013.
41. Feng, Y.; Yao, S.; Pu, Z.; Cheng, H.; Fei, B.; Zou, J.; Huang, Z. Identification of New Tumor-Related Gene Mutations in Chinese Gastrointestinal Stromal Tumors. *Frontiers in cell and developmental biology* **2021**, *9*, 764275, doi:10.3389/fcell.2021.764275.
42. Cui, S.; Fan, L.; Bai, Y.; Sun, X.; Cai, Y.; Dai, J.; Wang, T.; Sun, C.; Wang, R.; Liu, L. A case report of advanced small intestinal stromal tumor with KIT gene mutation and BRCA2 deletion after multi-line treatments. *Frontiers in oncology* **2025**, *15*, 1630699, doi:10.3389/fonc.2025.1630699.
43. Waisbren, J.; Uthe, R.; Siziopikou, K.; Kaklamani, V. BRCA 1/2 gene mutation and gastrointestinal stromal tumours: a potential association. *BMJ case reports* **2015**, *2015*, bcr2014208830, doi:10.1136/bcr-2014-208830.
44. Denu, R.A.; Joseph, C.P.; Urquiola, E.S.; Byrd, P.S.; Yang, R.K.; Ratan, R.; Zarzour, M.A.; Conley, A.P.; Araujo, D.M.; Ravi, V.; et al. Utility of Clinical Next Generation Sequencing Tests in KIT/PDGFRA/SDH Wild-Type Gastrointestinal Stromal Tumors. *Cancers* **2024**, *16*, 1707, doi:10.3390/cancers16091707.
45. Casetti, L.; Martin-Lannerée, S.; Najjar, I.; Plo, I.; Augé, S.; Roy, L.; Chomel, J.C.; Lauret, E.; Turhan, A.G.; Dusanter-Fourt, I. Differential contributions of STAT5A and STAT5B to stress protection and tyrosine kinase inhibitor resistance of chronic myeloid leukemia stem/progenitor cells. *Cancer research* **2013**, *73*, 2052-2058, doi:10.1158/0008-5472.Can-12-3955.
46. Nishida, T.; Naito, Y.; Takahashi, T.; Saito, T.; Hisamori, S.; Manaka, D.; Ogawa, K.; Hirota, S.; Ichikawa, H. Molecular and clinicopathological features of KIT/PDGFRA wild-type gastrointestinal stromal tumors. *Cancer science* **2024**, *115*, 894-904, doi:10.1111/cas.16058.
47. van der Wijngaart, H.; Beekhof, R.; Knol, J.C.; Henneman, A.A.; de Goeij-de Haas, R.; Piersma, S.R.; Pham, T.V.; Jimenez, C.R.; Verheul, H.M.W.; Labots, M. Candidate biomarkers for treatment benefit from sunitinib in patients with advanced renal cell carcinoma using mass spectrometry-based (phospho)proteomics. *Clinical proteomics* **2023**, *20*, 49, doi:10.1186/s12014-023-09437-6.
48. Chen, Z.H.; Qi, J.J.; Wu, Q.N.; Lu, J.H.; Liu, Z.X.; Wang, Y.; Hu, P.S.; Li, T.; Lin, J.F.; Wu, X.Y.; et al. Eukaryotic initiation factor 4A2 promotes experimental metastasis and oxaliplatin resistance in colorectal cancer. *Journal of experimental & clinical cancer research : CR* **2019**,

38, 196, doi:10.1186/s13046-019-1178-z.
49. Liu, M.; Gong, C.; Xu, R.; Chen, Y.; Wang, X. MicroRNA-5195-3p enhances the chemosensitivity of triple-negative breast cancer to paclitaxel by downregulating EIF4A2. *Cellular & molecular biology letters* **2019**, *24*, 47, doi:10.1186/s11658-019-0168-7.
50. Zhao, Z.; Zhang, G.; Li, W. Elevated Expression of ERCC6 Confers Resistance to 5-Fluorouracil and Is Associated with Poor Patient Survival in Colorectal Cancer. *DNA and cell biology* **2017**, *36*, 781-786, doi:10.1089/dna.2017.3768.
51. Chen, C.; Liu, H.; Li, Y.; Liu, J. Association of ERCC family mutations with prognosis and immune checkpoint inhibitors response in multiple cancers. *Scientific reports* **2023**, *13*, 13925, doi:10.1038/s41598-023-40185-7.
52. Xu, R.; Zhu, S.; Zhang, W.; Xu, H.; Tu, C.; Wang, H.; Wang, L.; He, N.; Liu, T.; Guo, X.; et al. A Dual Approach with Organoid and CRISPR Screening Reveals ERCC6 as a Determinant of Cisplatin Resistance in Osteosarcoma. *Advanced science (Weinheim, Baden-Wurttemberg, Germany)* **2025**, *12*, e2500632, doi:10.1002/advs.202500632.
53. Qi, L.; Zhou, B.; Chen, J.; Hu, W.; Bai, R.; Ye, C.; Weng, X.; Zheng, S. Significant prognostic values of differentially expressed-aberrantly methylated hub genes in breast cancer. *Journal of Cancer* **2019**, *10*, 6618-6634, doi:10.7150/jca.33433.
54. O'Sullivan, J.; Kothari, C.; Caron, M.C.; Gagné, J.P.; Jin, Z.; Nonfoux, L.; Beneyton, A.; Coulombe, Y.; Thomas, M.; Atalay, N.; et al. ZNF432 stimulates PARylation and inhibits DNA resection to balance PARPi sensitivity and resistance. *Nucleic acids research* **2023**, *51*, 11056-11079, doi:10.1093/nar/gkad791.
55. He, D.; Li, T.; Sheng, M.; Yang, B. Exonuclease 1 (Exo1) Participates in Mammalian Non-Homologous End Joining and Contributes to Drug Resistance in Ovarian Cancer. *Medical science monitor : international medical journal of experimental and clinical research* **2020**, *26*, e918751, doi:10.12659/msm.918751.
56. Mazza, F.; Dalfovo, D.; Bartocci, A.; Lattanzi, G.; Romanel, A. Integrative Computational Analysis of Common EXO5 Haplotypes: Impact on Protein Dynamics, Genome Stability, and Cancer Progression. *Journal of chemical information and modeling* **2025**, *65*, 3640-3654, doi:10.1021/acs.jcim.5c00067.
57. Liu, Z.; Jiang, H.; Lee, S.Y.; Kong, N.; Chan, Y.W. FANCM promotes PARP inhibitor resistance by minimizing ssDNA gap formation and counteracting resection inhibition. *Cell reports* **2024**, *43*, 114464, doi:10.1016/j.celrep.2024.114464.
58. Zhang, Y.; Li, J.; Zhou, Y.; Li, Z.; Peng, C.; Pei, H.; Zhu, W. And-1 Coordinates with the FANCM Complex to Regulate Fanconi Anemia Signaling and Cisplatin Resistance. *Cancer research* **2022**, *82*, 3249-3262, doi:10.1158/0008-5472.Can-22-0769.
59. Kifayat, K.; Singh, K.; Khan, M.; Razia, D.E.M.; Khan, S.; Dong, C.; Wang, L. Targeting MERTK tyrosine kinase: Virtual screening and molecular dynamics insights for anti-cancer drug development. *PloS one* **2025**, *20*, e0334106, doi:10.1371/journal.pone.0334106.
60. Paes Dias, M.; Tripathi, V.; van der Heijden, I.; Cong, K.; Manolika, E.M.; Bhin, J.; Gogola, E.; Galanos, P.; Annunziato, S.; Lieftink, C.; et al. Loss of nuclear DNA ligase III reverts PARP inhibitor resistance in BRCA1/53BP1 double-deficient cells by exposing ssDNA gaps. *Molecular cell* **2021**, *81*, 4692-4708, doi:10.1016/j.molcel.2021.09.005.
61. Ali, R.; Alabdullah, M.; Algethami, M.; Alblihy, A.; Miligy, I.; Shoqafi, A.; Mesquita, K.A.; Abdel-Fatah, T.; Chan, S.Y.; Chiang, P.W.; et al. Ligase 1 is a predictor of platinum resistance

and its blockade is synthetically lethal in XRCC1 deficient epithelial ovarian cancers. *Theranostics* **2021**, *11*, 8350-8361, doi:10.7150/thno.51456.
62. Bader, S.A.; Walker, M.; Harrison, D.J. A human cancer-associated truncation of MBD4 causes dominant negative impairment of DNA repair in colon cancer cells. *British journal of cancer* **2007**, *96*, 660-666, doi:10.1038/sj.bjc.6603592.
63. Dinis, J.; Silva, V.; Gromicho, M.; Martins, C.; Laires, A.; Tavares, P.; Rendeiro, P.; Torres, F.; Rueff, J.; Rodrigues, A. DNA damage response in imatinib resistant chronic myeloid leukemia K562 cells. *Leukemia & lymphoma* **2012**, *53*, 2004-2014, doi:10.3109/10428194.2012.681654.
64. Bara, T.; Jung, I.; Gurzu, S.; Kádár, Z.; Kövecsi, A.; Bara, T., Jr. Giant gastrointestinal stromal tumor of the stomach: a challenging diagnostic and therapeutically approach. *Romanian journal of morphology and embryology = Revue roumaine de morphologie et embryologie* **2015**, *56*, 1503-1506.
65. Li, B.; Brady, S.W.; Ma, X.; Shen, S.; Zhang, Y.; Li, Y.; Szlachta, K.; Dong, L.; Liu, Y.; Yang, F.; et al. Therapy-induced mutations drive the genomic landscape of relapsed acute lymphoblastic leukemia. *Blood* **2020**, *135*, 41-55, doi:10.1182/blood.2019002220.
66. Anurag, M.; Punturi, N.; Hoog, J.; Bainbridge, M.N.; Ellis, M.J.; Haricharan, S. Comprehensive Profiling of DNA Repair Defects in Breast Cancer Identifies a Novel Class of Endocrine Therapy Resistance Drivers. *Clinical cancer research : an official journal of the American Association for Cancer Research* **2018**, *24*, 4887-4899, doi:10.1158/1078-0432.Ccr-17-3702.
67. Kadioglu, O.; Elbadawi, M.; Fleischer, E.; Efferth, T. Identification of Novel Anthracycline Resistance Genes and Their Inhibitors. *Pharmaceuticals (Basel, Switzerland)* **2021**, *14*, 1051, doi:10.3390/ph14101051.
68. He, W.; Pang, L.; Gong, S.; Wang, X.; Hou, L. Nei Endonuclease VIII-like 2 Gene rs8191670 Polymorphism affects the Sensitivity of Non-small Cell Lung Cancer to Cisplatin by binding with MiR-548a. *Journal of Cancer* **2020**, *11*, 4801-4809, doi:10.7150/jca.47495.
69. Lai, H.H.; Hung, L.Y.; Yen, C.J.; Hung, H.C.; Chen, R.Y.; Ku, Y.C.; Lo, H.T.; Tsai, H.W.; Lee, Y.P.; Yang, T.H.; et al. NEIL3 promotes hepatoma epithelial-mesenchymal transition by activating the BRAF/MEK/ERK/TWIST signaling pathway. *The Journal of pathology* **2022**, *258*, 339-352, doi:10.1002/path.6001.
70. Wang, Y.; Xu, L.; Shi, S.; Wu, S.; Meng, R.; Chen, H.; Jiang, Z. Deficiency of NEIL3 Enhances the Chemotherapy Resistance of Prostate Cancer. *International journal of molecular sciences* **2021**, *22*, 4098, doi:10.3390/ijms22084098.
71. Orlikova-Boyer, B.; Lorant, A.; Gajulapalli, S.R.; Cerella, C.; Schnekenburger, M.; Lee, J.Y.; Paik, J.Y.; Lee, Y.; Siegel, D.; Ross, D.; et al. Antileukemic potential of methylated indolequinone MAC681 through immunogenic necroptosis and PARP1 degradation. *Biomarker research* **2024**, *12*, 47, doi:10.1186/s40364-024-00594-w.
72. Xin, J.; Zhang, H.; Yin, D.; An, N.; Chen, Y.; Xu, J.; Zhang, J.; Liu, Z.; Liu, Y.; Yin, W.; et al. A novel coumarin derivative DBH2 inhibits proliferation and induces apoptosis of chronic myeloid leukemia cells. *Genes & diseases* **2023**, *10*, 596-607, doi:10.1016/j.gendis.2022.08.021.
73. Kulkarni, S.; Seneviratne, N.; Tosun, Ç.; Madhusudan, S. PARP inhibitors in ovarian cancer: Mechanisms of resistance and implications to therapy. *DNA repair* **2025**, *149*, 103830, doi:10.1016/j.dnarep.2025.103830.
74. Sung, H.Y.; Han, J.; Chae, Y.J.; Ju, W.; Lee Kang, J.; Park, A.K.; Ahn, J.H. Identification of a novel PARP4 gene promoter CpG locus associated with cisplatin chemoresistance. *BMB reports*

2023, *56*, 347-352, doi:10.5483/BMBRep.2022-0202.

75. Kozono, D.; Hua, X.; Wu, M.C.; Tolba, K.A.; Waqar, S.N.; Dragnev, K.H.; Cheng, H.; Hirsch, F.R.; Mack, P.C.; Gray, J.E.; et al. Lung-MAP Next-Generation Sequencing Analysis of Advanced Squamous Cell Lung Cancers (SWOG S1400). *Journal of thoracic oncology : official publication of the International Association for the Study of Lung Cancer* **2024**, *19*, 1618-1629, doi:10.1016/j.jtho.2024.07.024.
76. Sever, T.; Kilicarslan, C.; Pehlivan, M.; Kaynar, L.; Yilmaz, M.; Eser, B.; Okan, V.; Kurnaz, F.; Cetin, M.; Pehlivan, S. Research on and clinical importance of duplications in various chromosomal regions in addition to Philadelphia chromosome in chronic myeloid leukemia. *Journal of B.U.ON. : official journal of the Balkan Union of Oncology* **2012**, *17*, 490-496.
77. Fu, Y.; Yang, B.; Cui, Y.; Hu, X.; Li, X.; Lu, F.; Qin, T.; Zhang, L.; Hu, Z.; Guo, E.; et al. BRD4 inhibition impairs DNA mismatch repair, induces mismatch repair mutation signatures and creates therapeutic vulnerability to immune checkpoint blockade in MMR-proficient tumors. *Journal for immunotherapy of cancer* **2023**, *11*, e006070, doi:10.1136/jitc-2022-006070.
78. Anurag, M.; Jaehnig, E.J.; Krug, K.; Lei, J.T.; Bergstrom, E.J.; Kim, B.J.; Vashist, T.D.; Huynh, A.M.T.; Dou, Y.; Gou, X.; et al. Proteogenomic Markers of Chemotherapy Resistance and Response in Triple-Negative Breast Cancer. *Cancer discovery* **2022**, *12*, 2586-2605, doi:10.1158/2159-8290.Cd-22-0200.
79. Xiao, Y.; Ni, M.; Zheng, Z.; Liu, Y.; Yin, M.; Mao, S.; Zhao, Y.; Tian, B.; Wang, L.; Xu, H.; et al. POLM variant G312R promotes ovarian tumorigenesis through genomic instability and COL11A1-NF-κB axis. *American journal of physiology. Cell physiology* **2024**, *327*, C168-c183, doi:10.1152/ajpcell.00025.2024.
80. Zhang, W.; Li, W.; Yin, C.; Feng, C.; Liu, B.; Xu, H.; Jin, X.; Tu, C.; Li, Z. PRKDC Induces Chemoresistance in Osteosarcoma by Recruiting GDE2 to Stabilize GNAS and Activate AKT. *Cancer research* **2024**, *84*, 2873-2887, doi:10.1158/0008-5472.Can-24-0163.
81. Zhao, P.; Yuan, F.; Xu, L.; Jin, Z.; Liu, Y.; Su, J.; Yuan, L.; Peng, L.; Wang, C.; Zhang, G. HKDC1 reprograms lipid metabolism to enhance gastric cancer metastasis and cisplatin resistance via forming a ribonucleoprotein complex. *Cancer letters* **2023**, *569*, 216305, doi:10.1016/j.canlet.2023.216305.
82. Zhang, P.; Zhang, Z.; Zhou, X.; Qiu, W.; Chen, F.; Chen, W. Identification of genes associated with cisplatin resistance in human oral squamous cell carcinoma cell line. *BMC cancer* **2006**, *6*, 224, doi:10.1186/1471-2407-6-224.
83. Viziteu, E.; Klein, B.; Basbous, J.; Lin, Y.L.; Hirtz, C.; Gourzones, C.; Tiers, L.; Bruyer, A.; Vincent, L.; Grandmougin, C.; et al. RECQ1 helicase is involved in replication stress survival and drug resistance in multiple myeloma. *Leukemia* **2017**, *31*, 2104-2113, doi:10.1038/leu.2017.54.
84. Harvey-Jones, E.; Raghunandan, M.; Robbez-Masson, L.; Magraner-Pardo, L.; Alaguthurai, T.; Yablonovitch, A.; Yen, J.; Xiao, H.; Brough, R.; Frankum, J.; et al. Longitudinal profiling identifies co-occurring BRCA1/2 reversions, TP53BP1, RIF1 and PAXIP1 mutations in PARP inhibitor-resistant advanced breast cancer. *Annals of oncology : official journal of the European Society for Medical Oncology* **2024**, *35*, 364-380, doi:10.1016/j.annonc.2024.01.003.
85. Guidi, L.; Pellizzari, G.; Tarantino, P.; Valenza, C.; Curigliano, G. Resistance to Antibody-Drug Conjugates Targeting HER2 in Breast Cancer: Molecular Landscape and Future Challenges. *Cancers* **2023**, *15*, 1130, doi:10.3390/cancers15041130.

86. Engel, J.L.; Zhang, X.; Wu, M.; Wang, Y.; Espejo Valle-Inclán, J.; Hu, Q.; Woldehawariat, K.S.; Sanders, M.A.; Smogorzewska, A.; Chen, J.; et al. The Fanconi anemia pathway induces chromothripsis and ecDNA-driven cancer drug resistance. *Cell* **2024**, *187*, 6055-6070, doi:10.1016/j.cell.2024.08.001.
87. Zhao, X.; Feng, S.; Nitie, X.; Muluo, S.; Lei, Y. SLX1 silencing overcomes Olaparib resistance in metastatic castration-resistant prostate cancer by disrupting SLX4-mediated DNA repair complexes. *Cancer biology & therapy* **2025**, *26*, 2545062, doi:10.1080/15384047.2025.2545062.
88. Cao, J.; Wei, J.; Yang, P.; Zhang, T.; Chen, Z.; He, F.; Wei, F.; Chen, H.; Hu, H.; Zhong, J.; et al. Genome-scale CRISPR-Cas9 knockout screening in gastrointestinal stromal tumor with Imatinib resistance. *Molecular cancer* **2018**, *17*, 121, doi:10.1186/s12943-018-0865-2.
89. Song, B.; Yang, P.; Zhang, S. Cell fate regulation governed by p53: Friends or reversible foes in cancer therapy. *Cancer communications (London, England)* **2024**, *44*, 297-360, doi:10.1002/cac2.12520.
90. Mirza-Aghazadeh-Attari, M.; Mohammadzadeh, A.; Yousefi, B.; Mihanfar, A.; Karimian, A.; Majidinia, M. 53BP1: A key player of DNA damage response with critical functions in cancer. *DNA repair* **2019**, *73*, 110-119, doi:10.1016/j.dnarep.2018.11.008.
91. Schouten, P.C.; Vollebergh, M.A.; Opdam, M.; Jonkers, M.; Loden, M.; Wesseling, J.; Hauptmann, M.; Linn, S.C. High XIST and Low 53BP1 Expression Predict Poor Outcome after High-Dose Alkylating Chemotherapy in Patients with a BRCA1-like Breast Cancer. *Molecular cancer therapeutics* **2016**, *15*, 190-198, doi:10.1158/1535-7163.Mct-15-0470.
92. Cheng, W.H.; von Kobbe, C.; Opresko, P.L.; Fields, K.M.; Ren, J.; Kufe, D.; Bohr, V.A. Werner syndrome protein phosphorylation by abl tyrosine kinase regulates its activity and distribution. *Molecular and cellular biology* **2003**, *23*, 6385-6395, doi:10.1128/mcb.23.18.6385-6395.2003.
93. Picco, G.; Cattaneo, C.M.; van Vliet, E.J.; Crisafulli, G.; Rospo, G.; Consonni, S.; Vieira, S.F.; Rodríguez, I.S.; Cancelliere, C.; Banerjee, R.; et al. Werner Helicase Is a Synthetic-Lethal Vulnerability in Mismatch Repair-Deficient Colorectal Cancer Refractory to Targeted Therapies, Chemotherapy, and Immunotherapy. *Cancer discovery* **2021**, *11*, 1923-1937, doi:10.1158/2159-8290.Cd-20-1508.
94. Mi, L.; Cai, Y.; Qi, J.; Chen, L.; Li, Y.; Zhang, S.; Ran, H.; Qi, Q.; Zhang, C.; Wu, H.; et al. Elevated nonhomologous end-joining by AATF enables efficient DNA damage repair and therapeutic resistance in glioblastoma. *Nature communications* **2025**, *16*, 4941, doi:10.1038/s41467-025-60228-z.
95. Xu, M.; Huang, X.; Zheng, C.; Long, J.; Dai, Q.; Chen, Y.; Lu, J.; Pan, C.; Yao, S.; Li, J. Platinum-Resistant Ovarian Cancer Is Vulnerable to the cJUN-XRCC4 Pathway Inhibition. *Cancers* **2022**, *14*, 6068, doi:10.3390/cancers14246068.
96. Zhang, R.; Hao, J.; Yu, H.; Wang, Z.J.; Lan, F.; Peng, Y.; Qiu, Y. circ_SIRT1 upregulates ATG12 to facilitate Imatinib resistance in CML through interacting with EIF4A3. *Gene* **2024**, *893*, 147917, doi:10.1016/j.gene.2023.147917.
97. Dai, H.; Wang, J.; Huang, Z.; Zhang, H.; Wang, X.; Li, Q.; Feng, W. LncRNA OIP5-AS1 Promotes the Autophagy-Related Imatinib Resistance in Chronic Myeloid Leukemia Cells by Regulating miR-30e-5p/ATG12 Axis. *Technology in cancer research & treatment* **2021**, *20*, 15330338211052150, doi:10.1177/15330338211052150.
98. Chen, Y.; Wang, R.; Huang, S.; Henson, E.S.; Bi, J.; Gibson, S.B. Erb-b2 Receptor Tyrosine

Kinase 2 (ERBB2) Promotes ATG12-Dependent Autophagy Contributing to Treatment Resistance of Breast Cancer Cells. *Cancers* **2021**, *13*, 1038, doi:10.3390/cancers13051038.

99. Yu, Y.; Yang, L.; Zhao, M.; Zhu, S.; Kang, R.; Vernon, P.; Tang, D.; Cao, L. Targeting microRNA-30a-mediated autophagy enhances imatinib activity against human chronic myeloid leukemia cells. *Leukemia* **2012**, *26*, 1752-1760, doi:10.1038/leu.2012.65.
100. Yue, P.; He, Y.; Zuo, R.; Gong, W.; Wang, Y.; Chen, L.; Luo, Y.; Feng, Y.; Gao, Y.; Liu, Z.; et al. CCDC34 maintains stemness phenotype through β-catenin-mediated autophagy and promotes EGFR-TKI resistance in lung adenocarcinoma. *Cancer gene therapy* **2025**, *32*, 104-121, doi:10.1038/s41417-024-00843-y.
101. Chen, W.; Li, Z.; Liu, H.; Jiang, S.; Wang, G.; Sun, L.; Li, J.; Wang, X.; Yu, S.; Huang, J.; et al. MicroRNA-30a targets BECLIN-1 to inactivate autophagy and sensitizes gastrointestinal stromal tumor cells to imatinib. *Cell death & disease* **2020**, *11*, 198, doi:10.1038/s41419-020-2390-7.
102. Zhang, J.; Liu, X.; Yin, C.; Zong, S. hnRNPK/Beclin1 signaling regulates autophagy to promote imatinib resistance in Philadelphia chromosome-positive acute lymphoblastic leukemia cells. *Experimental hematology* **2022**, *108*, 46-54, doi:10.1016/j.exphem.2022.01.004.
103. Cao, H.X.; Miao, C.F.; Sang, L.N.; Huang, Y.M.; Zhang, R.; Sun, L.; Jiang, Z.X. Circ_0009910 promotes imatinib resistance through ULK1-induced autophagy by sponging miR-34a-5p in chronic myeloid leukemia. *Life sciences* **2020**, *243*, 117255, doi:10.1016/j.lfs.2020.117255.
104. Han, S.H.; Korm, S.; Han, Y.G.; Choi, S.Y.; Kim, S.H.; Chung, H.J.; Park, K.; Kim, J.Y.; Myung, K.; Lee, J.Y.; et al. GCA links TRAF6-ULK1-dependent autophagy activation in resistant chronic myeloid leukemia. *Autophagy* **2019**, *15*, 2076-2090, doi:10.1080/15548627.2019.1596492.
105. Xie, Q.; Lin, Q.; Li, D.; Chen, J. Imatinib induces autophagy via upregulating XIAP in GIST882 cells. *Biochemical and biophysical research communications* **2017**, *488*, 584-589, doi:10.1016/j.bbrc.2017.05.096.
106. Roy, R.; Paul, T.; Das, P.K.; Sinha, S.; Ray, S.S.; Bhattacharyya, M.; Biswas, N. ERK-mTOR crosstalk suppresses autophagy and upregulates proteasomal degradation pathway to confer chronic myeloid leukemia cells resistant to imatinib. *Experimental hematology* **2025**, 105330, doi:10.1016/j.exphem.2025.105330.
